# Thermoresponsive Gels Based on Cross-Linked Polymer-Grafted Cellulose Nanocrystals

**DOI:** 10.1021/acs.biomac.5c02262

**Published:** 2026-03-20

**Authors:** Matilde Folkesson, Justus Paul Wesseler, Carolina Pierucci, Chris Rader, Christoph Weder, Alessandro Ianiro, José Augusto Berrocal

**Affiliations:** † 311305 Adolphe Merkle Institute, Chemin des Verdiers 4, 1700 Fribourg, Switzerland; ‡ National Center for Competence in Research (NCCR) Bio-Inspired Materials, 27211 University of Fribourg, Chemin des Verdiers 4, 1700 Fribourg, Switzerland; § Laboratory for Biomimetic Membranes and Textiles, Empa, 111825 Swiss Federal Laboratories for Materials Science and Technology, Lerchenfeldstrasse 5, 9014 St. Gallen, Switzerland; ∥ Dep. of Chemistry, Catholic University of Leuven, Celestijnenlaan 200F, 3001 Leuven, Belgium; ⊥ Institute of Chemical Research of Catalonia (ICIQ), Barcelona Institute of Science and Technology (BIST), Avda. Països Catalans 16, 43007 Tarragona, Spain; # Catalan Institution for Research and Advanced Studies (ICREA), Pg. Lluís Companys 23, 08010 Barcelona, Spain

## Abstract

Stimuli-responsive nanocomposite hydrogels have garnered significant interest as alternatives to conventional hydrogels, enabling the engineering of stimuli-responsive behavior and network connectivity through composition and architecture. Here, we report thermoresponsive, “one-component” nanocomposite hydrogels composed of copolymer-grafted cellulose nanocrystals (CNCs). Thermoresponsive polyacrylamides or poly­(oligoethylene glycol acrylate) copolymers bearing terminal olefin side chains were *grafted from* the CNC surfaces using atom-transfer radical polymerization, yielding densely grafted hairy nanoparticles (HNPs). The HNPs were cross-linked via UV-mediated thiol–ene click chemistry to form hydrogels. The resulting networks exhibit reversible LCST-type swelling and deswelling, with thermoresponsive and mechanical behavior governed by graft chemistry, architecture, and solvation. Comparative experiments using CNC-free and physically mixed hydrogels show that, at the low CNC loadings employed here, mechanical properties are shaped predominantly by chain entanglement and solvation, rather than by reinforcement from the nanocrystals.

## Graphical abstract

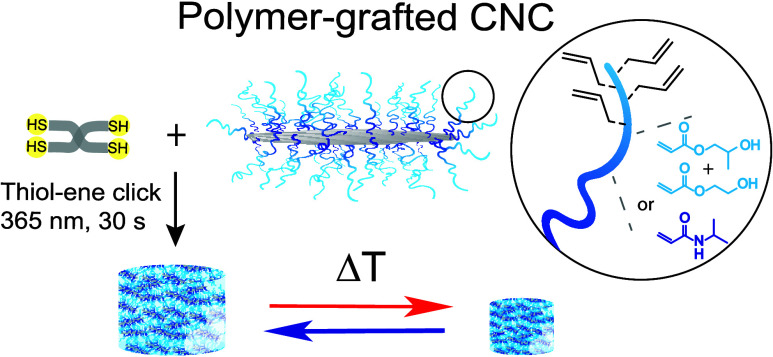

## Introduction

Nature has developed numerous systems that respond intelligently and reversibly to their environment; examples range from pinecones that open and close in response to changes in humidity to muscles that contract upon electrical stimulation.^1^ Translating these concepts into synthetic systems has led to the emergence of stimuli-responsive materials that can adapt their properties in response to environmental cues.^2–7^ Among them, hydrogels, i.e., three-dimensional polymer networks containing a large fraction of water,^8–10^ are particularly attractive due to their tunable physicochemical properties, making them suitable for applications such as tissue engineering, drug delivery, soft robotics, and actuation.^11–17^

Thermoresponsive hydrogels are especially useful because they respond to temperature changes through simple heating and cooling. In this context, poly­(*N*-isopropylacrylamide) (PNIPAm) and poly­(oligoethylene glycol acrylates) (POEGAs) are widely used because they display lower critical solution temperature (LCST) phase transitions.^18–20^ When the temperature of aqueous solutions exceeds or falls below the LCST, the polymer chains collapse or elongate upon expulsion or adsorption of solvent molecules.^19,21^

A typical drawback of conventional hydrogels is their low toughness, which is about two orders of magnitude lower than that of natural tissues.^22–24^ Strategies to overcome this include (i) enhancing cross-link homogeneity, (ii) introducing energy dissipation mechanisms, and (iii) incorporating multifunctional cross-linking points.^23,24^ Incorporating nanofillers addresses (iii) and has proven effective for reinforcing and toughening hydrogels.^13,25,26^

Cellulose nanocrystals (CNCs) are especially attractive as nanofillers due to their readily available surface groups, inherent mechanical strength, high crystallinity, aspect ratio, biocompatibility, and low cost.^27,28^ Stimuli-responsive CNC-composite hydrogels have found applications in self-healing, drug delivery, wound healing, piezoionic, and soft actuator materials, to name but a few.^29–36^ Their performance, however, is susceptible to how the CNCs are integrated, whether by physical mixing, chemical grafting, or network-forming incorporation.^37^

Common strategies for incorporating CNCs into hydrogel matrices typically include physical mixing of CNCs with the polymer network or chemical conjugation with the hydroxyl (–OH) groups present on the CNC surfaces.^13,37–39^ While physical mixing reduces synthetic complexity, it can result in phase separation when CNCs exhibit poor compatibility with the polymer matrix or the solvent.^40^ Therefore, covalent attachment of polymers to the CNC surface can be advantageous. Among surface grafting approaches, *grafting from* methodologies, wherein polymer chains are polymerized directly from the CNC surface, offer superior surface coverage and uniformity compared to *grafting to* strategies.^41,42^

A standard approach to make hydrogels comprising polymer-grafted CNCs involves free-radical polymerization from CNC surfaces, combined with simultaneous *in situ* cross-linking.^38,39,43–48^ These strategies yield covalently cross-linked materials but typically lack control over the polymer composition and molecular weight distribution, which are critical parameters for biomedical applications.^49–51^ In contrast, surface-initiated controlled radical polymerization (SI-CRP) techniques, among which surface-initiated atom transfer radical polymerization (SI-ATRP), yield grafted chains with a defined composition and architecture.^52^ This approach enables the synthesis of hairy nanoparticles (HNPs), facilitating the preparation of nanocomposite materials while preventing the aggregation or percolation of CNCs.^53–55^ Recent Scheutjens-Fleer self-consistent field theoretical models further suggest that materials based on covalently cross-linked HNPs could display promising mechanical and thermoresponsive properties.^56^ Moreover, solid, non-cross-linked “one-component” nanocomposites prepared from polymer-grafted CNCs have been shown to exhibit markedly different mechanical properties compared to analogous two-component systems. This divergence has been attributed to bottlebrush-like architecture and high entanglement density at low CNC contents.^55,57^

While the use of SI-CRP to decorate CNCs with stimuli-responsive polymers has been described in several reports,^58–65^ most hydrogels based on cross-linked HNPs rely on *physical* interactions. Li et al. and Azzam et al. formed hydrogels from CNCs grafted with thermoresponsive PNIPAm and Jeffamine, respectively.^66,67^ Gelation of the resulting HNPs was triggered by heating the aqueous dispersions above the LCST of the polymers, which led to the aggregation of the hydrophobic HNPs and the formation of physical hydrogels. The gels could easily be disassembled by lowering the temperature below the LCST.^66,67^ Finally, we highlight the *supramolecular cross-linking* approach based on dynamic cucurbit[8]­uril host–guest chemistry, employed by Scherman and Ikkala and co-workers.^68^ The authors created robust, self-healing nanocomposite hydrogels using polymer-grafted cellulose nanocrystals functionalized with cucurbit[8]­uril.^68^

Physically cross-linked hydrogels exhibit reversible sol–gel transitions but may lack structural integrity over a range of temperatures, a characteristic that can be imparted by *covalent* cross-links. Among potential cross-linking strategies, thiol–ene click chemistry offers a mild, rapid, and orthogonal method for cross-linking under UV light.^69^

To the best of our knowledge, thermoresponsive hydrogels formed by covalently linking CNCs grafted with cross-linkable thermoresponsive polymer brushes via SI-CRP have not been reported. The fundamental interest in such an approach derives from the precise composition of the polymer-grafted CNCs and their dual role as structural elements and cross-linkable building blocks. Here, we demonstrate the synthetic feasibility and examine how graft chemistry, cross-linking, and solvent interactions influence the mechanical and responsive behavior of these hydrogels, compared to reference CNC-free and physically mixed hydrogels. Our results define key design parameters for future development of one-component nanocomposite hydrogels.

## Experimental Section

### Materials

Acetone, allyl methacrylate (AMA), ascorbic acid, copper wire (ø = 1 mm), copper­(II) bromide (CuBr_2_), dialysis tube (12–14 kDa molecular weight cutoff (MWCO) membrane), diethyl ether, dry 1,4-dioxane, dry dimethyl sulfoxide (DMSO), dry dimethylformamide (DMF), ethanol (EtOH), α-bromoisobutyryl bromide (BiBB), ethyl bromoisobutyrate (EtBiB), hexane, 2-hydroxy-4′-(2-hydroxyethoxy)-2-methylpropiophenone (Irgacure 2959), 2-hydroxyethyl 2-bromoisobutyrate (HOBiB), hydroxyethyl acrylate (HEA), hydroxypropyl acrylate (HPA), methanol (MeOH), *N*-isopropylacrylamide (NIPAm), *N*,*N*-dimethylpyridin-4-amine (DMAP), *N*,*N*,*N*′,*N*″,*N*″-pentamethyldiethylenetriamine (PMDETA), pentaerythritol tetrakis­(3-mercaptopropionate) (PTM), pentenoic acid, sulfuric acid (95–97%), tetrahydrofuran (THF), triethylamine (TEA), tris­[2-(dimethylamino)­ethyl]­amine (Me_6_TREN) and, Whatman filter paper grade 1 were all purchased from Sigma-Aldrich. *N*-(2-Hydroxyethyl)­acrylamide (NHEAm) was purchased from TCI Chemicals. *N*-(3-(Dimethylamino)­propyl)-*N*′-ethylcarbodiimide hydrochloride (EDC·Cl) was purchased from Apollo Scientific. NIPAm was purified by recrystallization in hexane and stored at 4 °C prior to use. Before polymerization, the inhibitor was removed from NHEAm and AMA by passing the monomer over a plug of basic alumina (activated, 58 Å pore size), and from HEA and HPA by passing the monomer over neutral alumina (activated, 60 Å pore size).

### Synthetic Procedures

#### Extraction of Cellulose Nanocrystals (CNCs)

CNCs were extracted from Whatman filter paper by H_2_SO_4_ hydrolysis, following a reported procedure.^70^ Filter paper (30 g) was cut into pieces (<1 cm^2^) and gradually added to a 55 °C sulfuric acid solution (400 g, 64%). The mixture was stirred using a PTFE screw propeller-equipped rod. After 30 min, the reaction was quenched by adding 1000 mL of deionized water (DI H_2_O). The diluted mixture was centrifuged (20,000*g*, 20 min, 10 °C) using a Beckman Coulter Avanti J-26 XP centrifuge with a JLA-16.250 rotor (Indiana, US), and the supernatant was decanted. The CNC pellet was redispersed in DI H_2_O, and the process was repeated three times. The final pellet (white) was dialyzed against DI H_2_O (12–14 kDa MWCO membrane) for a week with water changes every 12 h. The resulting suspension (pH 7) was then lyophilized (−42 °C, 0.4 mbar) using a Telstar Lyoquest (Terrassa, Spain), resulting in a white powder (10.9 g).

#### Functionalization of CNCs with ATRP Initiators (**CNC-Br**)

**CNC-Br** was synthesized following the procedure reported by Zhang et al.^71^ Lyophilized CNCs (1000 mg) dispersed in dry DMF (100 mL) were added to a 250 mL Schlenk flask equipped with a stirring bar. The mixture was sonicated in a water bath (15 °C) for 1 h. Under an N_2_ atmosphere, TEA (57.4 mmol, 8 mL) and DMAP (32.8 mmol, 4 g) were added. The mixture underwent three vacuum-N_2_ cycles. The Schlenk flask was immersed in an ice bath, and BiBB (64.8 mmol, 8 mL) was added dropwise while stirring. The reaction proceeded overnight at room temperature, under N_2_. The reaction was quenched with ethanol (200 mL), and the resulting suspension was centrifuged (at 8000*g* for 10 min) using a Jouan B4i centrifuge (Massachusetts, US) with a Thermo AB 50.10A rotor. The pellet was redispersed in acetone and centrifuged. The procedure was repeated three times. The product was dialyzed against DI H_2_O (12–14 kDa MWCO membrane) for a week with water changes every 12 h. The purified product was lyophilized (−42 °C, 0.4 mbar), resulting in a white powder with a yellow tint (2 g).

#### CNC-*g*-P­(NIPAm)-*grad*-P­(NHEAm) (**CGN**)

**CNC-Br** (201 mg, 0.69 mmol initiator) was added to a 50 mL Schlenk flask containing DMF (25 mL). The resulting mixture was subjected to ultrasound for 30–60 min. After a homogeneous dispersion of **CNC-Br** was obtained, NIPAm (12.2 g, 108 mmol), CuBr_2_ (15.5 mg, 0.07 mmol), Me_6_TREN (45 μL, 0.17 mmol) were added along with Milli-Q water (MQ H_2_O) (100 mL) to reach a 1:4 v/v DMF/H_2_O solvent mixture and a **CNC-Br** dispersion of 0.19%w/v. The flask was sealed with a septum, immersed in an ice bath and sparged with argon for 20 min. In two separate glass vials with pierceable membrane caps, ascorbic acid (3 mg, 0.017 mmol) was dissolved in MQ H_2_O (1 mL), and NHEAm (11.2 mL, 108 mmol) was dissolved in MQ H_2_O (12 mL). Both solutions were sparged with argon for 20 min. The ascorbic acid solution was slowly added to the Schlenk flask using an argon-flushed syringe. The addition of ascorbic acid initiated the polymerization. After 10 min, the NHEAm solution was transferred to the reaction flask using an argon-flushed syringe. The final reaction contained a molar ratio of 155:155:1:0.1:0.25:0.025 of [NIPAm]/[NHEAm]/[**CNC-Br**]/[CuII]/[Me_6_TREN]/[AscA], and an initial concentration of monomer [M]_0_ of 1.03 M prior to the addition of NHEAm. The polymerization was allowed to proceed overnight with magnetic stirring, during which the reaction mixture became highly viscous. The P­(NIPAm)-*grad*-P­(NHEAm) grafted CNCs were isolated and purified by centrifugation (8000*g*, 10 min) three times with acetone and once with DI H_2_O. Residual water was removed by freeze-drying. **CGN** was obtained as a white, powder (9.6 g, 75%).

#### Saponification of CGN for SEC Analysis

Saponification of **CGN** was performed to cleave the grafted P­(NIPAm)-*grad*-P­(NHEAm) chains. The procedure was adapted from Zoppe et al.^59^ Briefly, 50 mg of **CGN** was dispersed in 20 mL of a 2 wt % NaOH aqueous solution and stirred for 48 h at room temperature. Following saponification, the dispersion was neutralized with 6 N HCl and centrifuged (8000*g*, 5 min) to separate the CNCs from the cleaved polymer chains in the supernatant. The supernatant was collected and dialyzed against DI H_2_O (12–14 kDa MWCO membrane) until a minimum in conductivity was reached. The recovered polymer was freeze-dried prior to SEC analysis.

#### CNC-*g*-P­(NIPAm)-*grad*-P­(NHEAm-*stat*-AllylAcrylamide) (**CGN-A**)

P­(NIPAm)-*grad*-P­(NHEAm) (8.4 g) was added to a round-bottom flask (RBF) containing DMF (80 mL) and was sonicated for 30 min. Pentenoic acid (4.75 mL, 46.5 mmol), EDC·Cl (7.22 g, 46.5 mmol), and DMAP (748 mg, 6.1 mmol) were added. The reaction mixture was stirred at room temperature overnight. The reaction mixture was diluted with ethanol, causing flocculation. The mixture was centrifuged (8000*g*, 10 min) and the solvent was decanted. The functionalized CNC-*g*-P­(NIPAm)-*grad*-P­(NHEAm-*stat*-AllylAcrylamide) was centrifuged (8000*g*, 10 min) three times in ethanol, once in DI H_2_O, and was thereafter freeze-dried. **CGN-A** was obtained as a faintly yellow powder (6.2 g, 48%).

#### CNC-*g*-P­(HPA-*stat*-HEA)-*grad*-P­(AMA) (**CGO-A**)

**CNC-Br** (65 mg, 0.23 mmol initiator) was added to a 100 mL one-neck RBF containing a 2:1 v/v solution (29 mL) of dry DMSO and dry 1,4-dioxane. The resulting mixture was sonicated in a cooled bath (15 °C) for 1 h. After a homogeneous dispersion of **CNC-Br** was obtained, HPA (4.5 mL, 34 mmol), HEA (3.7 mL, 35 mmol), CuBr_2_ (5.1 mg, 0.23 mmol), and Cu(0) thread (12 cm—previously polished by sandpaper and twisted around a stir bar) were added to the flask to reach a **CNC-Br** dispersion of 0.18%w/v. The RBF was sealed using a silicon stopper, and the mixture was sparged with nitrogen (N_2_) via a needle while stirring for 1.5 h. The outlet needle was then removed while the inlet needle was maintained but was pulled up over the liquid phase to keep an N_2_ atmosphere. Me_6_TREN (14.75 μL, 0.057 mmol—previously sparged for 1 h with N_2_) was added under N_2_ to start the surface-initiated polymerization. The reaction mixture became viscous after 80 min. A 2:1 v/v solution (9 mL) of dry DMSO and dry 1,4-dioxane (previously sparged with N_2_ for 1 h) was added to the mixture via an N_2_-flushed syringe to dilute the reaction mixture. After 260 min, the 2:1 v/v mixture of dry DMSO and dry 1,4-dioxane previously sparged with N_2_ (10 mL) was added again under N_2_. A solution of dry DMSO (10 mL), CuBr_2_ (5.0 mg, 0.023 mmol), AMA (3 mL, 22.87 mmol), and Me_6_TREN (14.75 μL, 0.057 mmol) (previously sparged for 1 h with N_2_) was added via a N_2_ flushed syringe under N_2_ after 272 min, and the reaction flask was slowly heated to 70 °C. After 350 min, a 2:1 v/v solution (10 mL) of dry DMSO and dry 1,4-dioxane (previously sparged with N_2_ for 1 h) was added to the mixture via an N_2_-flushed syringe. The polymerization was stopped after 367 min by removing the silicon plug and venting the flask with air. The reaction mixture was diluted with acetone (50 mL) and centrifuged (8000*g*, 10 min). The resulting pellet was redispersed in acetone and centrifuged (8000*g*, 10 min) twice. The procedure was repeated with ethanol (×2) and, last, DI H_2_O (×2). The purified product was dispersed in DI H_2_O and sonicated in a cooled bath (15 °C) for 30 min. The dispersion was frozen and lyophilized at −42 °C and 0.4 mbar. **CGO-A** was obtained as a white spongy material (4.7 g, 89%).

#### P­(NIPAm-*stat*-NHEAm) (N)

NIPAm (2 g, 17.7 mmol) and NHEAm (0.98 mL, 9.5 mmol) were added to a 50 mL Schlenk flask fitted with a magnetic stirrer and dissolved in a DMF/H_2_O 3:7 v/v mixture. To this solution, HOBiB (17.2 μL, 0.12 mmol), CuBr_2_ (13.9 mg, 0.062 mmol), and Me_6_TREN (133 μL, 0.50 mmol) were added. The mixture was then submerged in an ice bath (0 °C) and sparged with dry N_2_ for 20 min while stirring. A 2.2 mg/mL (0.012 mmol/mL) stock solution of ascorbic acid was prepared and sparged with dry N_2_ for 20 min. After 20 min had passed, a plastic syringe purged with N_2_ was used to transfer 1 mL of the ascorbic acid stock solution into the Schlenk flask to initiate the polymerization. The reaction was allowed to proceed overnight at 0 °C, after which it was terminated by opening the flask to air and precipitating the crude product into hexane. To fully remove the copper catalyst and unreacted monomers, the crude polymer was redissolved in methanol (MeOH)/H_2_O 1:1 v/v, transferred to an RBF, and subsequently concentrated in vacuo at 60 °C. As the solvent was removed, the polymer began to precipitate and agglomerate. At this point, the flask was removed from the rotary evaporator, and the remaining solvent was decanted. This process was repeated at least once, yielding a transparent sticky mass. Drying in a vacuum oven overnight (80 °C) yielded the final polymer as a white powder (2.78 g).

#### P­(NIPAm-*stat*-(NHEAm-*stat*-AllylAcrylamide)) (N-A)

The nongrafted polymer **N** (2.52 g) was dissolved in DMF (25 mL) in a 50 mL RBF equipped with a magnetic stirrer. This amount of polymer corresponded to 7.76 mmol of NHEAm as determined by ^1^H NMR analysis. 4-Pentenoic acid (0.48 mL, 4.65 mmol), EDC·Cl (0.72 g, 4.65 mmol), and DMAP (110 mg, 0.90 mmol) were added sequentially to the mixture, and the reaction was allowed to proceed at room temperature overnight. The crude polymer was precipitated into diethyl ether and redissolved into MeOH a minimum of two times. Finally, the polymer was placed in a vacuum oven (80 °C) overnight to yield the final product as a yellow powder (2.42 g).

#### P­(HPA-*stat*-HEA) (O)

CuBr_2_ (17.5 mg, 0.8 mmol) was added to a 100 mL one-neck RBF with a 2:1 v/v solution (34 mL) of dry DMSO and dry 1,4-dioxane. HPA (18.4 mL, 140 mmol), HEA (15.2 mL, 140 mmol), and EtBiB (114.5 μL, 0.78 mmol) were added under air. A Cu(0) thread (20 cm—previously polished by sandpaper and twisted around a stir bar) was introduced. The RBF was sealed using a silicon stopper, and the mixture was sparged with N_2_ while stirring (1 h). The outlet needle was removed, and the inlet needle was adjusted above the liquid phase to maintain the N_2_ atmosphere. Me_6_TREN (50.6 μL, 0.20 mmol) pre-sparged with N_2_ (1 h) was added under N_2_ to initiate the polymerization. The mixture became viscous after 2 min, and the polymerization was quenched after 20 min by diluting it with ethanol (100 mL). The product was purified by passing it over silica and the solvent was evaporated. The concentrated product was precipitated in diethyl ether, redissolved in ethanol, and reprecipitated in diethyl ether. This precipitation procedure was repeated twice. The product was vacuum-dried (40 °C) overnight, yielding a transparent sticky film (18 g).

#### P­((HPA-*stat*-AllylAcrylate)-*stat*-(HEA-*stat*-AllylAcrylate)) (O-A)

The nongrafted polymer **O** (6.1 g) was added to a 100 mL one-neck RBF with dry DMF (60.5 mL), EDC·Cl (2.4 g, 12.5 mmol), and DMAP (0.19 g, 1.6 mmol). The RBF was sealed using a silicon plug and was sonicated in a bath for 5 min followed by sparging with N_2_ while stirring (1 h). The outlet needle was removed, and the inlet needle was adjusted above the liquid phase to maintain the N_2_ atmosphere. While stirring in a water bath, 4-pentenoic acid (850 μL, 8.4 mmol) was added dropwise under N_2_ (RT). The reaction mixture was stirred overnight. The solvent was removed. The concentrated product was precipitated in diethyl ether, redissolved in ethanol, and reprecipitated in diethyl ether. This precipitation procedure was repeated twice. The product was vacuum-dried (40 °C) overnight, yielding a yellow sticky film (5 g).

### Characterization

#### Transmission Electron Microscopy (TEM)

TEM images of CNCs and **CNC-Br** were obtained using a FEI/ThermoFisher Tecnai Spirit transmission electron microscope (Hillsboro, OR) equipped with a LaB6 emitter (120 kV) and a 2k Veleta camera. Pictures were taken at a spot size of 4 (CNCs) or 6 (**CNC-Br**), with a magnification of 20.5k×, and with a defocus of −10 μm. Samples (0.01%w/w CNCs in MQ H_2_O or 0.05%w/w **CNC-Br** in THF) were sonicated in a bath (15 °C) for 1 h, deposited (15 μL) on plasma-treated (Zepto Rie Diener electronic, 30 s at 50% power under O_2_) TEM grids (carbon film 300 mesh copper) placed on a filter paper, and dried overnight at room temperature before imaging. The dimensions of the CNCs and **CNC-Br** were measured using ImageJ/FIJI software.

#### Atomic Force Microscopy (AFM)

The average height of the CNCs was obtained from AFM images. Freshly peeled mica was coated with an aqueous solution of poly­(l-lysine) (0.1%w/v in H_2_O) by drop-casting. After 5 min, the excess poly­(l-lysine) was washed off with MQ H_2_O, and the substrates were dried under a flow of dry N_2_. A dispersion of the produced CNC (0.001%w/w) was then dropped-casted onto the functionalized mica surface. The images were acquired with a Park NX10 from Park Systems in tapping mode with a Tap300AI-G probe at room temperature.

#### Conductometric Titration

Conductometric titration was performed using a Mettler Toledo SevenCompact Duo with an InLab 731-ISM probe to determine the sulfate-half ester (R-OSO_3_H) content of CNCs by titrating against a 0.01 M NaOH solution. Prior to titration, CNCs were dialyzed in DI H_2_O for 6 days, with water exchange every 12 h, to remove residual sulfate ions. CNCs were then passed over a Dowex Marathon C hydrogen strong acid cation resin to ensure complete protonation of sulfate-half ester groups. The equivalence point was determined from the intersection of least-squares regression lines fitted to distinct regions of the titration curve. The titration was performed in triplicates, yielding an R-OSO_3_H concentration of 190 ± 1 mmol kg^–1^ (Figure S3).

#### Elemental Analysis (EA)

EA was performed by the Molecular and Biomolecular Analysis Service at ETH Zurich, Switzerland, to determine the carbon (C), hydrogen (H), oxygen (O), and bromine (Br) content in the lyophilized **CNC-Br**. The C and H content was measured using a LECO TruSpec CHN-micro (Michigan, US), which analyzes the combustion products (CO_2_ and H_2_O) from the sample digestion at 1050 °C by infrared spectrometry. The O content was determined using a LECO TruSpec CHN-O (Michigan, US) by pyrolyzing the sample in a pure He flow at 1300 °C with excess C. The resulting CO was oxidized to CO_2_ via CuO and measured using infrared spectroscopy. The Br content was determined by digesting the sample using the Schöniger method and analyzing the combustion product (HBr), captured in an absorbing solution, by ion chromatography. The determined content of each element by EA is the average of duplicates, C: 37.77 ± 0.16%w/w; H: 4.78 ± 0.06%w/w; O: 26.36 ± 0.3%w/w; Br: 27.57 ± 0.07%w/w.

#### Proton Nuclear Magnetic Resonance Spectroscopy (^1^H NMR)

^1^H NMR spectra were recorded at 297.2 K on a Bruker Avance DPX 400 spectrometer at 400.19 MHz. All NMR spectra were acquired in DMSO-*d*_6_, where polymer-grafted CNCs form stable dispersions over the time scale of the measurements. Chemical shifts (δ) were referenced to the residual solvent peak of DMSO-*d*_6_. Data analysis was performed using MestReNova software (v 11.0). All δ are reported in parts per million (ppm) relative to tetramethylsilane.

#### Size Exclusion Chromatography (SEC)

SEC was performed on an Agilent 1260 Infinity II HPLC system equipped with one Agilent PolarGel M guard column (8 μm) and two Agilent PolarGel M columns (7.5 mm × 300 mm, 8 μm). Samples (1.3 mg/mL) were eluted at 60 °C in DMF with 0.05 M LiBr at 1.0 mL/min. Molecular weights were determined using poly­(ethylene oxide) calibration standards.

#### Thermogravimetric Analysis (TGA)

TGA was performed using a Mettler-Toledo TGA/DSC 1 STAR System from 25 to 600 °C at a heating rate of 10 °C min^–1^ under nitrogen. Data were analyzed using the STARe software.

#### Attenuated Total Reflection Fourier Transmission Infrared Spectroscopy (ATR-FTIR)

ATR-FTIR spectra were recorded using a PerkinElmer Spectrum 65 (Shelton, CT) spectrometer with an ATR setup. Spectra were collected from 600 to 4000 cm^–1^ over 8 scans. The spectra were baseline corrected.

#### X-ray Diffraction (XRD)

XRD patterns were recorded on a Bruker D2 Phaser diffractometer using Cu Kα radiation (λ = 1.54184 Å, 30 kV, and 10 mA) with a 1.0 mm divergence slit and 2.5° Soller slit. Samples were analyzed as dried powders or polymer films on a steel sample holder. For each sample, data were collected over a 2θ range of 5–40°, with a step size of 0.01° and a counting time of 1 s per step. Due to the limited availability of the material, **CNC-Br** produced a lower signal-to-noise ratio. The raw data was converted using PowDLL and the data was background-subtracted by the sample holder spectrum.

#### Diffusing Wave Spectroscopy (DWS)

The LCST of the control polymers **N** and **O** (3% w/w in MQ H2O) was determined using a DWS-Rheolab instrument by monitoring scattering frequency changes between 30 and 90 °C. Measurements were performed in quartz cuvettes with a 10 mm optical path, detecting backscattered light in the VH configuration (vertically polarized laser, horizontally polarized scattered light). The measurements were taken with a 60 s repetition duration and a 60 s repetition duration echo, with three repetitions per temperature step. After each measurement, the solution was cooled to room temperature before the next temperature step. The LCST was estimated as the temperature at the derivate maximum of the scattering curve after a 3.5 min conditioning time. The LCST was 65 °C for N (Figure S16.A) and 56 °C for O (Figure S16.B).

### Gel Preparation and Characterization

#### Cross-Linking of **CGN-A** and **CGO-A** into **CGN-G** and **CGO-G**

Gels of **CGN-A** or **CGO-A** were formed by cross-linking the solvent-dispersed polymer-grafted CNCs with pentaerythritol tetrakis­(3-mercaptopropionate) (PTM) as a cross-linker, using Irgacure 2959 as photoinitiator upon irradiation with UV light (λ = 365 nm). The extent of the allyl-functional side chains in **CGN-A** and **CGO-A** was determined by ^1^H NMR spectroscopy (Figures 1A and S6) and amounted to 1.5 mmol/g and 0.8 mmol/g, respectively (calculations on pages S4–S5). The molar ratio of allyl units to PTM was set to [4:1] (*n*_PTM_ = *n*_Allyl_/4), and the molar ratio of allyl units to Irgacure 2959 was set to 1:0.04. A concentration of 18%w/v **CGN-A** or **CGO-A** in DMSO was determined to be useful for the preparation of gels, and the corresponding stock dispersions of **CGN-A** and **CGO-A** were prepared. Thus, **CGN-A** or **CGO-A** (0.5 g) and DMSO (2 mL) were added to 4 mL screw-cap vials. The dispersions were first mixed with a spatula, subsequently vortexed for 5 min, and thereafter sonicated in a cooled bath (15 °C) for about 1 h. The **CGN-A** mixture was further placed on a shaker overnight (250 rpm). The DMSO dispersions of **CGN-A**/**CGO-A** were mixed with PTM and Irgacure 2959 and were then filled into 2 mL plastic molds produced from syringes. The nozzle was sealed, and the vials were centrifuged (282 g, 2–5 min). This ensured the formation of air bubble-free homogeneous mixtures. The mixtures were photo-cross-linked in a Hönle LED cube 100 (Gräfelfing, Germany) UV chamber (365 nm at 180 mW cm^–2^) for 30 s to produce cylindrical **CGN-A**/**CGO-A** organogels (**CGN-G** and **CGO-G**, respectively).

**Figure 1:**
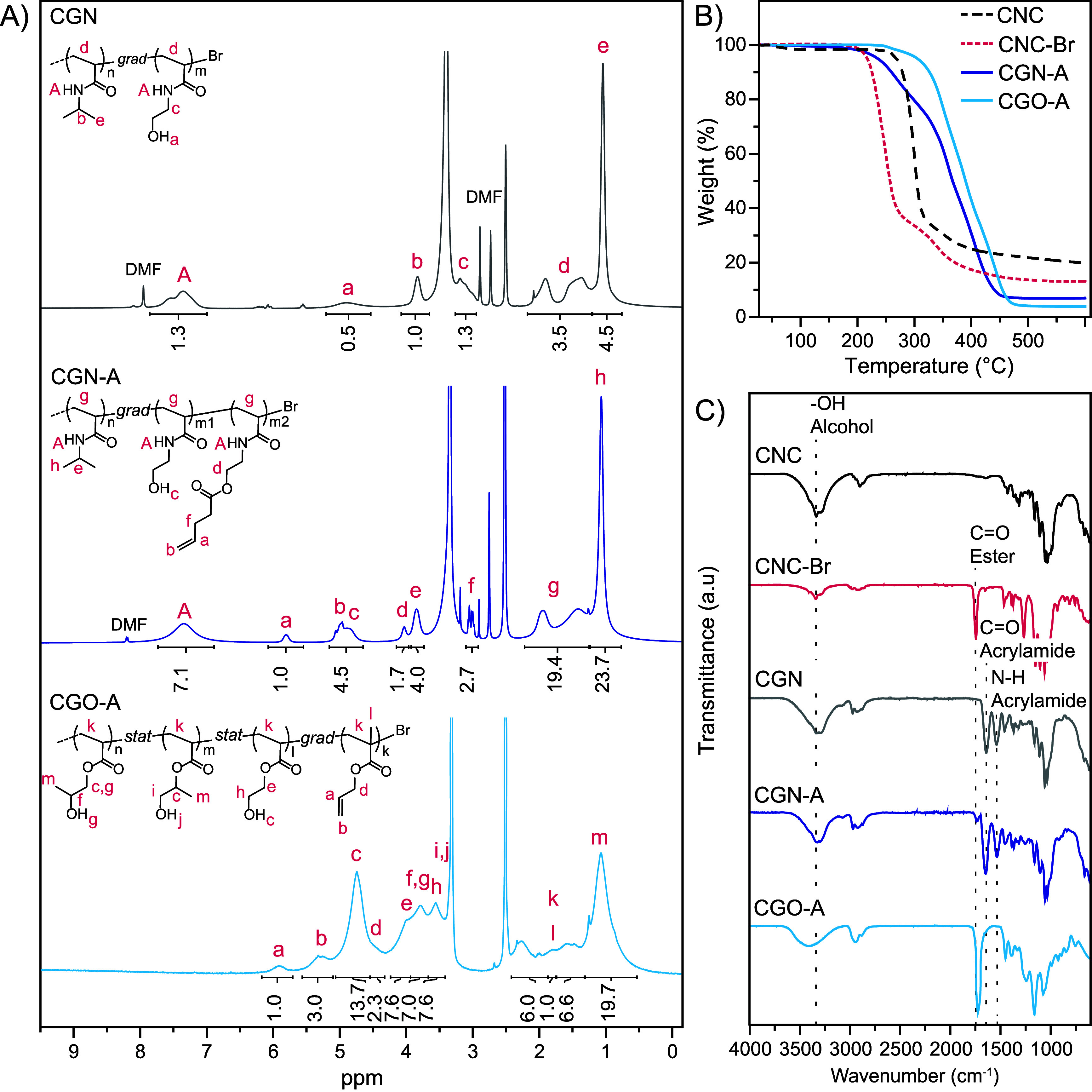
(A) ^1^H NMR (DMSO-d_6_, 400 MHz) spectra of **CGN**, **CGN-A**, and **CGO-A**. (B) TGA traces of pristine CNCs, initiator-functionalized CNCs (**CNC-Br**), **CGN-A**, and **CGO-A**. (C) FTIR spectra of pristine CNCs, **CNC-Br**, **CGN**, **CGN-A**, and **CGO-A**.

#### Cross-Linking of **N-A** and **O-A** to Gels

Gels of **N-A** and **O-A** were prepared as controls to the **CGN-G** and **CGO-G** using the same concentration of polymer in DMSO (18%w/v), molar ratio of allyl units to PTM [4:1] (*n*_PTM_ = *n*_Allyl_/4), and molar ratio of allyl units to Irgacure 2959 (1:0.04). The average amount of allyl-functional side chains in **N-**A (1.08 mmol/g) and **O-A** (1.28 mmol/g) was determined by ^1^H NMR spectroscopy as described on pages S4–S5.

To investigate the effect of CNC incorporation, two formulations were prepared: one with CNCs (**N-G**_**CNC**_ and **O-G**_**CNC**_) having the same concentration of CNCs as in **CGN-G** (0.17%w/v) and **CGO-G** (0.12%w/v) as calculated on page S6, and one with only polymer (**N-G**_**OP**_ and **O-G**_**OP**_). Unlike the paste-like consistency of the HNP precursor mixture, the mixtures of the polymer systems were more liquid-like. The mixtures were mixed by a spatula and vortex, then UV-irradiated while in cylindrical molds (365 nm, at 180 mW cm^–2^) for 30 s.

#### Gel Solvent Exchange

One batch of each gel was kept in DMSO, while another batch was solvent exchanged from DMSO to water. The samples kept in DMSO are hereafter referred to as “as-prepared”, while the term “solvent exchanged” is used for the samples obtained from the solvent exchange to DI H_2_O. The solvent exchange was carried out by immersing the DMSO gels in a 20 mL vial with DMSO and slowly exchanging the DMSO to water by diluting it with cold DI H_2_O at regular time intervals of 2 h over 12 h.

#### Water Contents in Gels

**CGN-G**, **N-G**, **CGO-G**, **O-G** solvent exchanged to DI H_2_O were dried at high air flow overnight. The mass of the dried gels was recorded (*n* = 3) and the water content was calculated as the ratio of the mass lost upon drying to the initial hydrated mass.

#### Rheological Measurements

Disks (ø = 8 mm, *h* ≈ 3–4 mm) of HNP gels (**CGN-G**, **CGO-G**) “as prepared” and “solvent exchanged” to DI H_2_O (*n* ≥ 5) underwent amplitude sweeps (10 rad/s, 0.01–100 strain %) using an MCR 702 Anton Paar Rheometer (Virginia, US) equipped with a profiled flat top and bottom geometry (ø = 8 mm and 25 mm, respectively). The storage and loss modulus (Pa) were determined from the linear viscoelastic region. The same protocol was used for the control gels **N-G** and **O-G**.

#### Compression Measurements

Disks (ø = 8 mm, *h* ≈ 3–4 mm) of HNP gels (**CGN-G**, **CGO-G**) solvent exchanged to DI H_2_O (*n* = 3) underwent compression testing using an MCR 702 Anton Paar Rheometer (Virginia, US) equipped with a profiled flat top and bottom geometry (ø = 8 mm and 25 mm, respectively). The gels were compressed until failure at a speed of 100 μm/s. The compressive Young’s modulus was obtained from the slope of the stress–strain curve’s initial linear region (1–6% strain). The same protocol was used for the control gels **N-G** and **O-G**.

#### Temperature Response Measurements

The temperature response of “solvent exchanged” **CGN-G** and **CGO-G** (*n* ≥ 4) was measured by subjecting the materials to steps of increasing temperatures to reach 5 °C, 21 °C, 40 °C, and 60 °C, and then cooled down to reach 40 °C, 21 °C, and 5 °C. The gels were equilibrated at each temperature for 5 min, after which the diameter and mass were measured. The gels were stored at 5 °C overnight, and the heating/cooling cycle was repeated. In total, three cycles were carried out. The same protocol was used for the control gels **N-G** and **O-G**.

## Results and Discussion

### System Design

The design of the materials departs from CNCs, which are functionalized with an ATRP initiator (as in **CNC-Br**) (Scheme 1A) that enables the *grafting from* reaction of thermoresponsive PNIPAm- or POEGA-based gradient copolymer chains. The former is a gradient copolymer of NIPAm and *N*–hydroxyethyl acrylamide (NHEAm) that was used to exploit the well-known LCST of PNIPAm (ca. 32 °C) and NHEAm’s hydroxyl group for postpolymerization modification. The POEGA used here is a statistical copolymer of hydroxyethyl acrylate (HEA) and hydroxypropyl acrylate (HPA) (75:25 mol/mol). HPA is a commercially available mixture of two isomers (vide infra) whose copolymerization with HEA displays “ideal random” copolymerization kinetics,^72^ affording copolymers with thermoresponsive properties that can be tuned by polymer composition.^72,73^ Grafting such copolymers from **CNC-Br** leads to **CGN** (PNIPAm-based HNP) or **CGO** (POEGA-based HNP). We targeted a high surface functionalization density of initiators, which in turn limits the CNC content in the resulting HNPs to approximately 1 wt %. This results in structural characteristics that are more reminiscent of bottlebrush polymers than rigid nanoparticles.^55,74^ We elected to employ postpolymerization modification of hydroxyl groups (in the case of **CGN**) and the copolymerization with allyl-bearing functional comonomers (in the case of **CGO**) to incorporate allyl moieties, affording **CGN-A** and **CGO-A** (Scheme 1A), respectively. Note that in both cases, the grafts exhibited a compositional gradient, with the allyl groups being preferentially incorporated toward the chain ends. We expected that cross-linking dispersions of such HNPs with pentaerythritol tetrakis­(3–mercaptopropionate) (PTM) via photostimulated thiol–ene click chemistry^69^ would afford the corresponding gels **CGN-G** and **CGO-G**. We demonstrate that these gels respond to temperature shifts by reversibly swelling/deswelling and identify key design parameters for future optimization, including graft length and density, allyl moiety content, and cross-linker stoichiometry during gel formation.

**Scheme 1:**
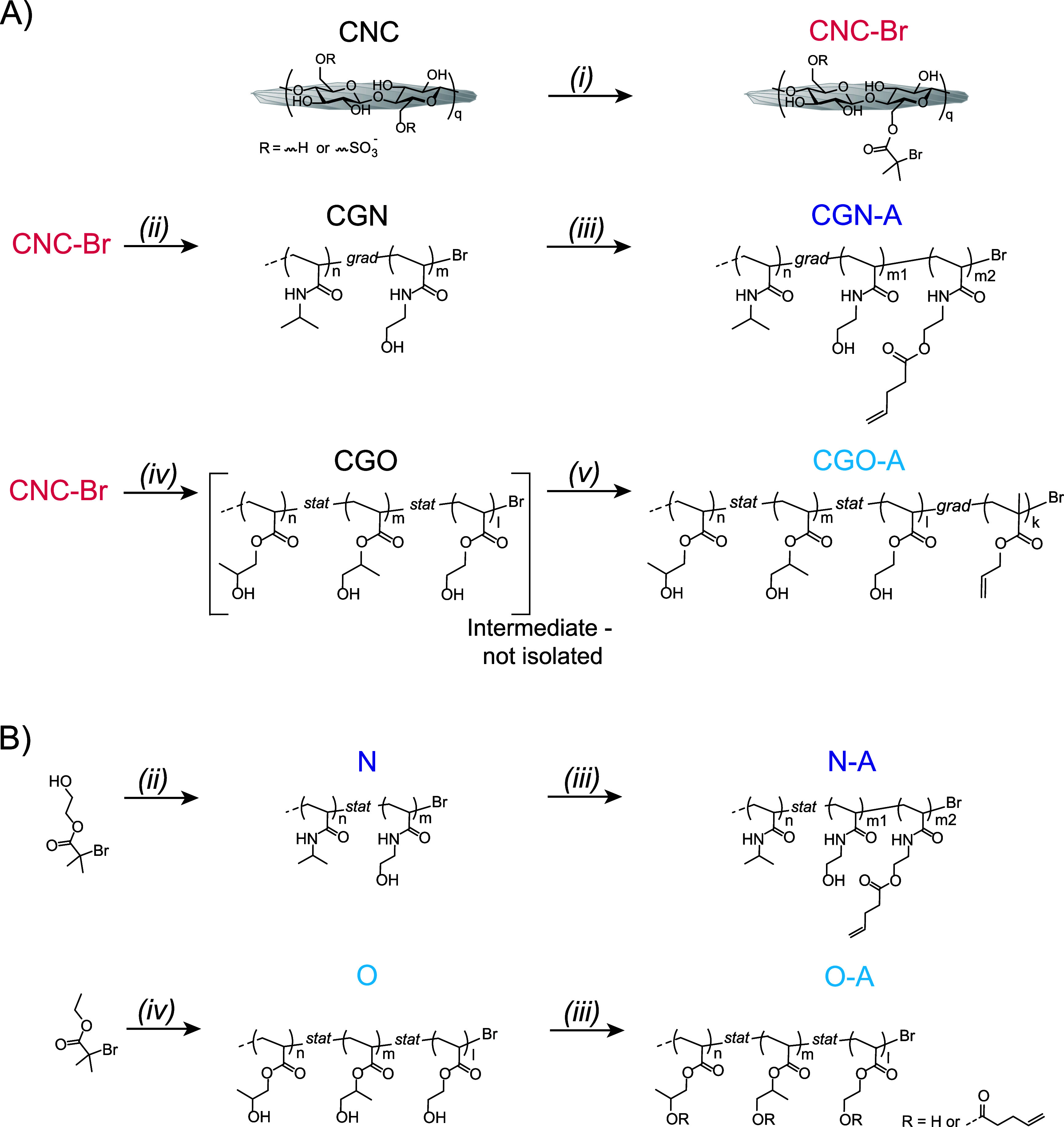
(A) Functionalization of CNCs with BiBB to Afford ATRP Macro Initiators **CNC-Br**. The **CNC-Br** were Grafted with Thermoresponsive Polymers PNIPAm and POEGA, Yielding **CGN** and **CGO** (Not Isolated), Respectively. **CGN** and **CGO** were Modified to Incorporate a Gradient of Crosslinkable Allyl Groups at the Chain-Ends, Affording **CGN-A** and **CGO-A**, Respectively. (B) Synthesis of Free PNIPAm and POEGA Control Polymers **N** and **O**, and Their Allyl Functionalized Counterparts **N-A** and **O-A**. Reaction Conditions (i) BiBB, DMAP, TEA, DMF, 0 °C to r.t., 18 h (ii) NIPAm, NHEAm, CuBr_2_, Me_6_TREN, Ascorbic Acid, DMF/H_2_O, 0 °C to r.t., 18 h (iii) Pentenoic Acid, EDC·Cl, DMAP, DMF, r.t., 18 h (iv) HPA, HEA, CuBr_2_, Me_6_TREN, Copper Wire, DMSO/1,4-Dioxane (2:1), r.t., 4.5 h (Grafted from Surface) or 20 min (Free Polymer), (v) AMA, CuBr_2_, Me_6_TREN, Copper Wire, DMSO, 70 °C, 1.5 h

### Synthesis and Characterization of Functionalized CNCs and Preparation of Gels

The CNCs were prepared by sulfuric acid hydrolysis of cotton-based paper, following a previously reported procedure.^75^ The dimensions of the unmodified CNCs were determined by transmission electron microscopy (TEM) and atomic force microscopy (AFM), which revealed an average length of 160 ± 40 nm (TEM), width of 12 ± 3 nm (TEM), height of 6 ± 1 nm (AFM), and an aspect ratio (L/W) of 14 ± 4 (TEM) (see Supporting Information (SI), Figures S1 and S2), i.e., dimensions that are typical for this type of CNC.^76,77^ Conductometric titrations determined the sulfate half-esters content of CNCs to be 190 ± 1 mmol kg^–1^ (Figure S3), which falls within the typical range of surface charge density for CNCs produced by sulfuric acid hydrolysis of cotton-derived cellulose.^78^

The CNCs were then functionalized with ATRP initiators by reacting the surface hydroxyl groups with α–bromoisobutyryl bromide (BiBB) according to an established method to afford **CNC-Br** (Scheme 1A).^55^ The TEM analysis of **CNC-Br** reveals an average length, width, and aspect ratio of 180 ± 50 nm, 11 ± 3 nm, and 18 ± 7, respectively (Figure S4). While the dimensions of the **CNC-Br** are similar to those of pristine CNCs, their morphology appears more frayed. The introduction of α–bromoisobutyryl groups was confirmed by elemental analysis (EA), which reveals a Br content of 27.6 ± 0.1%. This value is exceptionally high and exceeds the Br content of 15% previously reported by Majoinen et al.^79^ Such Br loading corresponds to a grafting density of ATRP initiators (σ_i_) of 12.6 nm^–2^ (further discussion on the determination of σ_i_ on page S3). We speculate that the exceptionally high Br content might derive from the frayed morphology of **CNC-Br**, which allows more hydroxyl groups to be exposed and, hence, functionalized.

With **CNC-Br** in hand, we proceeded to graft thermoresponsive PNIPAm and POEGAs from their surface (Scheme 1A). As discussed above, different approaches were used to incorporate a gradient of cross-linkable allyl moieties toward the end of the polymer chains. In the PNIPAm system, we performed a copolymerization with a gradient of NHEAm, which provided the hydroxyl groups to introduce the allyl moieties in **CGN-A** via postmodification (Scheme 1A). For **CGO-A**, we introduced a gradient of allyl methacrylate (AMA) moieties directly into the POEGA backbone, instead (Scheme 1A).

The SI-ATRP of NIPAm (with 50 mol % NHEAm, which was added 10 min after the polymerization of NIPAm was initiated; see Experimental Section) from **CNC-Br** was carried out in a 4:1 v/v DI H_2_O/DMF mixture. DMF ensured the effective dispersion of **CNC-Br**, which could not be dispersed in DI H_2_O alone. The choice of the mixed solvent was supported by previous literature, which suggests that water-assisted ATRP of acrylamides in DI H_2_O/DMF solvent mixtures is characterized by good control over polymer dispersity (*Đ*) and polymerization kinetics.^80^ A drawback of the ATRP of NIPAm is the possibility of intramolecular cyclization of the growing PNIPAm chains.^81^ This process can be mitigated by carrying out the polymerization at low temperature,^81^ which is the rationale for our choice to conduct the SI-ATRP of NIPAm at approximately 0 °C. The *grafting from* procedure to produce **CGN** (Scheme 1A) was facilitated by surface-initiated activator-generated electron transfer (SI-AGET) ATRP approach, in which ascorbic acid serves as the reducing agent for the oxidatively stable CuBr_2_.

Recent studies have revealed that densely polymer-grafted HNPs can be characterized by ^1^H NMR solution spectroscopy, which provides a valuable tool for following the **CNC-Br**-initiated polymerizations.^55^ We briefly introduce the use of this technique here, although its implementation will be elaborated further below. Initial investigations focused on the ^1^H NMR monitoring of the **CNC-Br**-initiated ATRP of NIPAm alone. These experiments revealed that ca. 61% NIPAm conversion was attained within 10 min under the following conditions: 155:1:0.1:0.25:0.025 molar ratio of [NIPAm]/[**CNC-Br**]/[CuII]/[ligand]/[AscA], 0 °C, and [M]_0_ = 1.03 M. The theoretical number-average molecular weight (*M*_*n*_) of these grafted chains was determined by multiplying the calculated number-average degree of polymerization (DP) by the molar mass of NIPAm, yielding a theoretical *M*_*n*_ of ca. 12,400 g/mol (Figure S5; for the method, see page S4). We deemed such conversion and *M*_*n*_ sufficient to prepare the targeted HNPs. Thus, the **CNC-Br**-initiated ATRP of NIPAm was repeated, and NHEAm (50 mol %) was added to the reaction mixture 10 min after starting the polymerization to access HNPs with gradient copolymer grafts. The polymerizations were allowed to proceed for 5 h at 0 °C and then stirred overnight to slowly allow the reaction mixtures to reach room temperature. This temperature profile was chosen because, in some cases, extended polymerization at 0 °C resulted in highly viscous (gel-like) reaction mixtures that prevented maximal monomer conversion. **CGN** was isolated through multiple centrifugation steps. The hydroxyl units of **CGN** were subsequently functionalized to introduce allyl units for thiol–ene cross-linking by EDC-mediated esterification with 4–pentenoic acid (Scheme 1A). This synthetic sequence afforded **CGN-A** with a yield of 48%; the loss of ca. 50% of the material is likely attributable to the increased dispersibility of the allyl-functionalized species in the organic solvents used during purification, which limits efficient pelleting during centrifugation, causing material loss during supernatant removal.

The SI–ATRP of POEGAs from **CNC-Br** to produce **CGO-A** was performed in a 2:1 v/v DMSO/1,4–dioxane solvent mixture (Scheme 1A). DMSO effectively disperses **CNC-Br** and serves as an alternative to DMF. Moreover, we found that 1,4–dioxane improves the solubility of CuBr_2_ in the presence of the monomers.^82^ The polymerization method was based on a previously reported procedure that used Cu(0) wire and CuBr_2_/Me_6_TREN (Scheme 1A).^55^ The comproportionation of the two Cu species generates Cu­(I) *in situ*, which initiates the polymerization. Using this method, statistical copolymers of HPA and HEA were grafted from the surface of the CNCs at room temperature, generating P­(HPA-*stat*-HEA) (**CGO**) (Scheme 1A). Also in this case, the polymerization mixture became highly viscous over time (gel-like), likely due to polymer chain entanglements among the HNPs. Thus, small portions of the solvent mixture (10 mL every 1.5–3 h) were added gradually over time to maintain the ability to magnetically stir the reaction mixture (Figure S6). After 4.5 h, allyl methacrylate (AMA) was added to the polymerization mixture (Scheme 1A). The reaction temperature was raised to 70 °C to ensure the polymerization of the less reactive methacrylate-based AMA comonomer in **CGO-A**. The mixture was stirred for an additional 1.5 h, after which the polymerization was quenched by adding acetone. The crude material was purified by repeated washing and centrifugation with acetone, ethanol, and water, and was thereafter lyophilized to provide **CGO-A**.

We also synthesized non-CNC-grafted PNIPAm (**N**) and POEGA (**O**), having a similar monomer composition as the grafted macromolecules, and allyl side-chain containing P­(NIPAm-*stat*-(NHEAm-*stat*-AllylAcrylamide)) (**N-A**) and P­((HPA-*stat*-AllylAcrylate)-*stat*-(HEA-*stat*-AllylAcrylate)) (**O-A**) to make control systems (Scheme 1B). For the synthetic procedures of the control polymers **N**, **N-A**, **O**, and **O-A**, see the Experimental Section. The ^1^H NMR spectra of colloidally stable dispersions of **CGN**, **CGN-A**, and **CGO-A** in DMSO-*d*_*6*_ provide insights into the composition of the grafted polymers (Figure 1A). The integration of the signals of the hydroxyl proton of NHEAm at 4.90 ppm (protons *a*) and of the isopropyl groups of NIPAm at 3.83 ppm (protons *b*) in the ^1^H NMR spectrum of **CGN** affords a molar NIPAm/NHEAm ratio of 65:35, i.e., the NHEAm fraction (35 mol %) in **CGN** is somewhat lower than in the feed (50 mol %). As discussed above, the NIPAm conversion reached ca. 61% after 10 min under our experimental conditions, and the polymers display a broadened molecular weight distribution after this reaction time, which suggests that some loss of living chain ends occurred prior to NHEAm introduction. The formation of gel-like mixtures also yielded a distinctly heterogeneous mixture in comparison to the initial dispersion of **CNC-Br** and monomers. All these factors might be responsible for the discrepancy between the monomer feed ratio and the final polymer composition in **CGN**. We calculated a total conversion of 51.6%, corresponding to individual conversions of 67% and 36% for NIPAm and NHEAm, respectively. Following purification, a yield of 75% was recovered. The CNC content in dried **CGN** was calculated to be ca. 1.0 wt% (see pages S3–S4). As mentioned before, this very low CNC content bestows the HNPs with a structure that can be considered more bottlebrush- than nanoparticle-like.

The fraction of allyl side chains in **CGN-A** was quantified by comparing the integral of the peaks at 5.81 ppm (protons *a*), characteristic of the pendant allyl groups, and the one at 3.83 ppm ascribed to the isopropyl groups of NIPAm (protons *e*), with the integral of the signal at 7.35 ppm corresponding to the amide protons of all monomer units (protons *A*) (Figure 1A). Since the signals of the allyl groups overlap with the characteristic hydroxyl signal of NHEAm (protons *c*), the fraction of NHEAm in **CGN-A** was determined as the remainder of unaccounted acrylamide (page S4). This analysis reveals that NIPAm, NHEAm, and AllylAm fractions comprise 65, 18, and 17 mol %, respectively, suggesting that half of the original NHEAm groups in **CGN** were converted into allyl groups. The composition of the nongrafted model-polymer **N-A** is very similar, with NIPAm, NHEAm, and allyl fractions of 65, 22, and 13 mol %, respectively (Figure S7 and page S4).

In the case of **CGO-A**, the ^1^H NMR spectra reveal several overlapping peaks due to the structural similarity of HPA and HEA residues (Figure 1A). This situation is further complicated by the fact that HPA is commercially sold as a mixture of two isomers, i.e., 2–hydroxypropyl acrylate and 1–methyl–2–hydroxyethyl acrylate, in a molar ratio of 75:25.^72,73^ Nevertheless, as HPA and HEA have been shown to have the same polymerization kinetics,^72^ it is reasonable to assume that the composition of the HNP grafts mirrors the one of the feed.^63^ The conversion was determined by integrating two sets of characteristic peaks and averaging the result. The acrylate proton signal at 6.32 ppm (proton *f*_*m*_) was compared to the polymer backbone signals between 2.30 and 1.20 ppm (protons *f*_*p*_), while signals at 4.84 ppm (protons *c*_*m*_), originating from the HPA and HEA side chains, were compared to their polymerized counterparts at 4.74 ppm (protons *c*_*p*_) (Figure S8). This analysis reveals a final conversion of 52% at 6 h. The fraction of AMA residues in the grafts can be calculated from the ^1^H NMR data in two ways, (i) from the conversion at the final reaction time (6 h) (Figure S6 and page S5), or (ii) from the ratio of the sum of the allyl group signals at 5.92 ppm (proton *a*, *I* = 1) and 5.30 ppm (protons *b*, *I* = 3.0) associated with HPA residues at 1.05 ppm (protons *m*, *I* = 19.7) in the purified and lyophilized product (Figure 1A). The AMA conversion, calculated as the average of both methods, was determined to be 20% (20 units, 0.9 mmol/g). Collectively, the analysis of the ^1^H NMR data affords HPA, HEA, and AMA fractions of 44, 45, and 11 mol %, respectively. Following purification, the product was isolated with an 89% yield. The CNC content in dried **CGO-A** was calculated to be 0.7 wt % (see page S6), indicating that the particles adopt a bottlebrush structure, as in the **CGN-A** system. The nongrafted model-polymer **O-A** has a similar composition, with HPA, HEA, and allyl fractions of 42.5, 42.5, and 15 mol %, respectively (Figure S8 and page S5).

While ^1^H NMR spectroscopy allowed the determination of the composition of the polymer chains in **CGN-A** and **CGO-A**, the determination of the *M*_*n*_ of the polymer chains grafted via SI–ATRP was carried out with two different methods. For the **CGN** system (i.e., before functionalization with allyl groups), the P­(NIPAm-*grad*-NHEAm) grafts were cleaved from the CNCs by saponification, and their dispersity and molecular weight were determined by size exclusion chromatography (SEC) (see Experimental Section). The SEC chromatograms reveal an *M_n_* of 13,600 and a *Đ* of 1.58, suggesting that the SI–ATRP polymerization that produced **CGN-A** suffered a loss in control compared to the nongraft solution ATRP counterpart **N-A** (18,600 g/mol and *Đ* = 1.20) (Figure S9.A). This difference in control can be rationalized by established literature on SI-ATRP, which shows that high grafting densities tend to accelerate polymerization kinetics due to increased local initiator concentration and restricted chain mobility at the surface.^83^ Nevertheless, we emphasize that this is a qualitative, literature-supported explanation offered to contextualize the broader dispersity of the grafted chains in **CGN-A**.

The *M_n_* and control over the polymerization of the **CGO-A** HNPs were assessed using ^1^H NMR spectroscopy instead, since saponification, in addition to cleaving off the polymer, would also hydrolyze the ester side chains. The theoretical *M*_*n*_ of the grafted polymer chains in **CGO-A** was assessed to be 22,800 g/mol and was determined from the conversion calculated by ^1^H NMR (see page S4). Additionally, the slope of the logarithm of monomer concentration versus reaction time was found to be relatively linear (Figure S9.B), suggesting that the polymerization was controlled. This can be compared with the nongraft control system **O-A**, which, from SEC analysis, was determined to have an *M*_*n*_ of 23,040 g/mol and *Đ* = 1.23 (Figure S9.C).

The CNC-derived species and nongrafted control polymers were further characterized by thermogravimetric analysis (TGA). The TGA trace of pristine CNCs reveals a slight loss in mass between 25–100 °C, primarily due to the evaporation of water (Figure 1B). This is followed by a main degradation process characterized by an onset at 263 °C, a maximum temperature of 318 °C, and a mass loss of 62% (Figure 1B), which is typical of unmodified CNCs.^84^ Further degradation led to additional mass loss and a char yield of 20% at 600 °C (Figure 1B). The introduction of ATRP initiator groups resulted in a TGA profile that is significantly different from that of pristine CNCs (Figure 1B). In line with previous reports,^55,85^ the main degradation onset of **CNC-Br** occurs at 200 °C (Figure 1B), indicating that the initiator functionalization reduces the thermal stability of **CNC-Br**, possibly due to the evolution of HBr that accelerates the degradation.^71^ The reduced char yield (13% at 600 °C), compared to that of neat CNCs (20%), is consistent with the high level of surface functionalization.

The TGA trace of **CGN-A** (Figure 1B) reveals a two-stage degradation process; the first step ranges from 200 to 320 °C and is associated with a 25% mass loss, while the second step spans from 320 to 450 °C and corresponds to a 66% mass loss. A comparison between the TGA traces of **CGN-A** and the nongrafted model-polymer **N-A** shows that the degradation profiles are similar, albeit with slight differences in onset temperatures and weight losses (Figure S10). This similarity suggests that the thermal stability of **CGN-A** is largely determined by the polymer component rather than the cellulose core, consistent with the high polymer content (ca. 99 wt %) in **CGN-A**. We attributed the first weight loss to the decomposition of the hydroxyethyl side chain of the NHEAm units (and the corresponding ethyl pent–4–enoate side chain, when present), followed by the decomposition of the acrylamide polymer.^86^ Note that the char yield (7%) is much lower than that of CNC and **CNC-Br**, once again reflecting that the HNPs consist almost entirely of polymer.

The TGA profile of **CGO-A** displays an even higher stability than **CGN-A**, with a one-step degradation process with an onset temperature of 320 °C and a total weight loss of 96% (Figure 1B). A comparison between the TGA traces of **CGO-A** and nongrafted model-polymer **O-A** shows that the degradation profiles are similar, with a slightly earlier onset temperature and steeper degradation curve for **O-A**, indicating that the thermal behavior of the HNPs is dominated by the grafted polymer chains (Figure S10).

Finally, FTIR spectroscopy also provided clear evidence of the transformation from CNC to **CNC-Br**, and from **CNC-Br** to **CGO-A** or **CGN-A**, based on signals in the spectral region between 1600 and 1800 cm^–1^, where carbonyl (C=O) stretching vibrations typically resonate (Figure 1C). The FTIR spectrum of pristine CNC is silent in that region, while a band centered at 1734 cm^–1^ emerged in the FTIR spectrum of **CNC-Br**, which corresponds to the C=O stretching vibration of the α–bromo ester groups introduced on the surface of the CNCs. Upon the introduction of the polymer chains of **CGN**, the peak at 1734 cm^–1^ disappears and is replaced by signals centered at 1640 cm^–1^ and 1539 cm^–1^, which are characteristic of C=O amide I and NH amide II stretching vibrations, respectively (Figure 1C). The presence of these peaks supports the successful grafting of polyacrylamides. Moreover, the increased intensity of the broad −OH peak centered at 3300 cm^–1^ confirmed the incorporation of NHEAm units in the polymer chains. Following partial functionalization of NHEAm units with pentenoic acid in **CGN-A**, a new peak is visible at 1732 cm^–1^, which can be assigned to the C=O stretching vibration of the newly formed ester bonds. Similar conclusions can be drawn from the FTIR spectrum of **CGO-A** (Figure 1C), which shows an intense signal at 1725 cm^–1^ that we attributed to the C=O stretching of the acrylate ester moieties and a broad peak at 3100–3700 cm^–1^ characteristic of O–H vibrations.

To examine whether the CNC crystalline structure was retained in the CNC → **CGN/CGN-A**/**CGO-A** series of transformations, we performed X-ray diffraction (XRD) analysis. The comparison of the XRD patterns of pristine CNCs, **CNC–Br**, and **CGN/CGN-A**/**CGO-A** speaks to a drastic loss of crystallinity upon surface functionalization (Figure S11.A). The characteristic cellulose I reflections of CNCs are notably broadened and less intense in **CNC–Br** (Figure S11.A), in line with TEM observations of a disrupted morphology for **CNC–Br**. Moreover, the XRD patterns of **CGN**/**CGN-A**/**CGO-A** are nearly indistinguishable from those of the corresponding free polymers **N**/**N-A** **O-A** (Figure S11.B,C), supporting the conclusion that the final HNPs are compositionally and structurally dominated by polymer. As such, any diffraction generated by crystalline CNC domains is too weak to be detectable.

With adequately characterized **CGN-A** and **CGO-A** HNPs in hand, we set out to prepare gels by cross-linking dispersions of these building blocks through UV-mediated thiol–ene click chemistry (Figure 2A).^69^ We screened conditions to achieve rapid and reproducible gelation using pentaerythritol tetrakis­(3-mercaptopropionate) (PTM) as a cross-linker (Figure 2A). A 1:1 molar ratio between allyl and thiol groups was selected to ensure consistent network formation and enable direct comparison between the two HNP systems. We reiterate that the fraction of allyl groups differs between **CGN-A** (17 mol %) and **CGO-A** (11 mol %) by approximately 50%; the PTM concentration was therefore adjusted accordingly to maintain the same effective allyl/thiol stoichiometry. We found that a 0.04:1 mol ratio of the photoinitiator Irgacure 2959 with respect to allyl groups resulted in rapid cross-linking of the precursor mixture.

**Figure 2:**
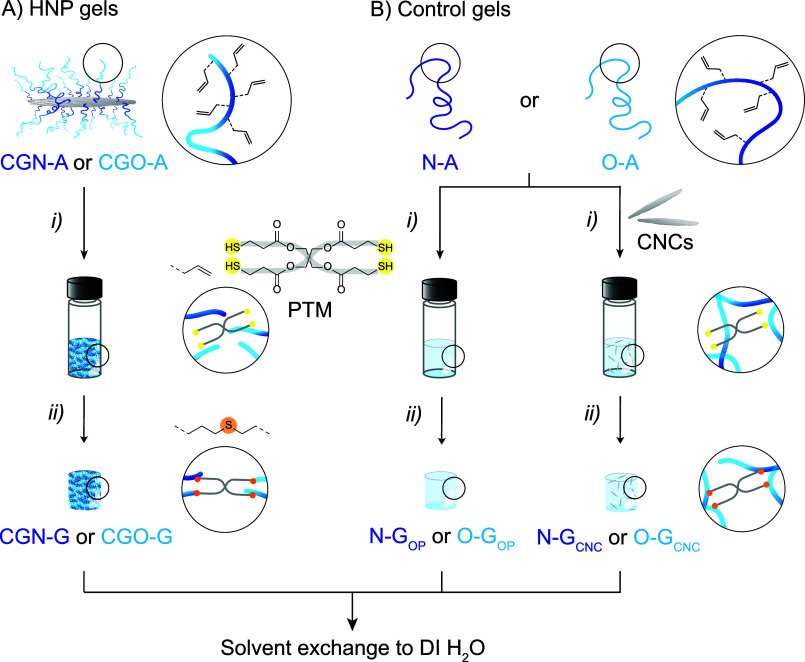
Schematic of the cross-linking process used to convert (A) **CGN-A** and **CGO-A** dispersions in DMSO into **CGN-G** and **CGO-G** organogels, and (B) the control polymers **N-A** in DMSO into **N-G**_**CNC**_ (adding CNCs) and **N-G**_**OP**_ organogels, and **O-A** in DMSO into **O-G**_**CNC**_ (with CNCs) and **O-G**_**OP**_ organogels. All gels were further converted to hydrogels after a solvent exchange with DI H_2_O. Reaction conditions: (i) mixing with DMSO, PTM, and Irgacure 2959, (ii) UV light (365 nm) for 30 s.

The ideal concentration of **CGN-A** and **CGO-A** HNPs in the pre-gel dispersion was determined by studying dispersions in DMSO in which the HNP content was varied from 8 to 22% w/v. Highly concentrated dispersions (>20%w/v) led to poor mixing, inefficient cross-linking, and inhomogeneous gels, while at lower concentrations (<16%w/v) the gels did not maintain their shape after removal from the mold. For both **CGN-A** and **CGO-A**, a concentration of 18%w/v in DMSO was found to be optimal to make **CGN-G** and **CGO-G** gels within 30 s of UV-induced thiol–ene cross-linking (Figure 2A). We note that at 18% w/v HNP content, the CNC concentration in the precursor dispersions is only ∼0.12–0.17% w/v (see SI, page S6). The precursor mixtures had a paste-like consistency that exhibited viscous flow prior to UV irradiation, but they turned into self-supported gels after cross-linking. Solvent exchange from DMSO to water (Figure 2A) resulted in an increased opacity and shrinking for both **CGN-G** and **CGO-G**. We speculate that the unreacted hydrophobic allyl groups in the grafted polymer chains and the dense packing of the polymer chains in the gels likely limit swelling and, therefore, result in opacity (Figure S12.A).

The nongrafted reference polymers **N-A** and **O-A** were photo-cross-linked into control **N-G** and **O-G** organogels using the same experimental conditions to make **CGN-G** and **CGO-G** (see Experimental Section) (Figure 2B). To investigate the effect of incorporating CNCs on the mechanical and thermoresponsive properties, we further synthesized two nanocomposite gels, namely (i) **N-G**_**CNC**_ and **O-G**_**CNC**_, which contained 1.0 or 0.7 wt % physically dispersed CNCs to match the CNC weight fractions of **CGN-G** and **CGO-G**, and (ii) **N-G**_**OP**_ and **O-G**_**OP**_, with only cross-linked polymer. The precursor mixtures of the control gels were more liquid-like, in contrast to the paste-like texture of the HNP precursor mixtures. However, they turned into self-standing gels after cross-linking, similarly to the HNP gels. The control gels also shrank upon solvent exchange, with **O-G** exhibiting a qualitatively stronger tendency to shrink compared to **N-G**, which collectively resulted in a lower water content for all **O-G** control gels (Figure S12.B). However, in stark contrast with the HNP gels, **N-G** and **O-G** control gels did not become opaque (Figure S12.A).

### Characterization of the Mechanical Properties and Thermoresponsive Behavior of the Gels

We then proceeded with the rheological characterization of **CGN-G** and **CGO-G**. Amplitude strain sweeps were conducted on both as-prepared organogels (Figure S13.A,B) as well as hydrogels prepared by solvent-exchange (Figure 3A,B). The storage modulus (*G*′), determined within the linear viscoelastic region, of the two DMSO organogels was similar, with values of 15.3 kPa for **CGN-G** and 13.3 ± 2.5 kPa for **CGO-G** organogel, which, in light of the higher allyl group and cross-linker concentration in **CGN-G**, is initially surprising. However, both HNPs form highly entangled physical networks during gel preparation, and this limits either the extent of cross-linking or the influence of cross-linking on the final gels’ properties. Interestingly, after solvent exchange with water, the *G*′ of the **CGN-G** hydrogel *decreased* to 9.8 ± 2.0 kPa, while *G*′ of the **CGO-G** hydrogel *increased* to 25.6 ± 3.0 kPa. Although both **CGN-G** and **CGO-G** became opaque (Figure S12.A) and shrank to some extent in water, **CGO-G** stiffened, whereas **CGN-G** surprisingly softened. A plausible explanation for this discrepancy is that the distinct chemical nature of the grafted chains—and in particular the ability of PNIPAm, unlike the POEGA used in this study, to engage in intra- and interchain hydrogen bonding—may influence chain hydration, mobility, and local polymer–solvent interactions. Although our experimental data do not allow us to conclusively assign a single mechanistic origin to this divergence, the observed differences underscore the importance of graft chemistry in dictating mechanical response and point to a compelling avenue for future study. Finally, we note that some slipping occurred at the highest shear strains (>10%) during rheological measurements, despite consistently using a crosshatched top plate to prevent wall slipping. Thus, conclusions about the position of the crossover point are marred by some uncertainty.

**Figure 3:**
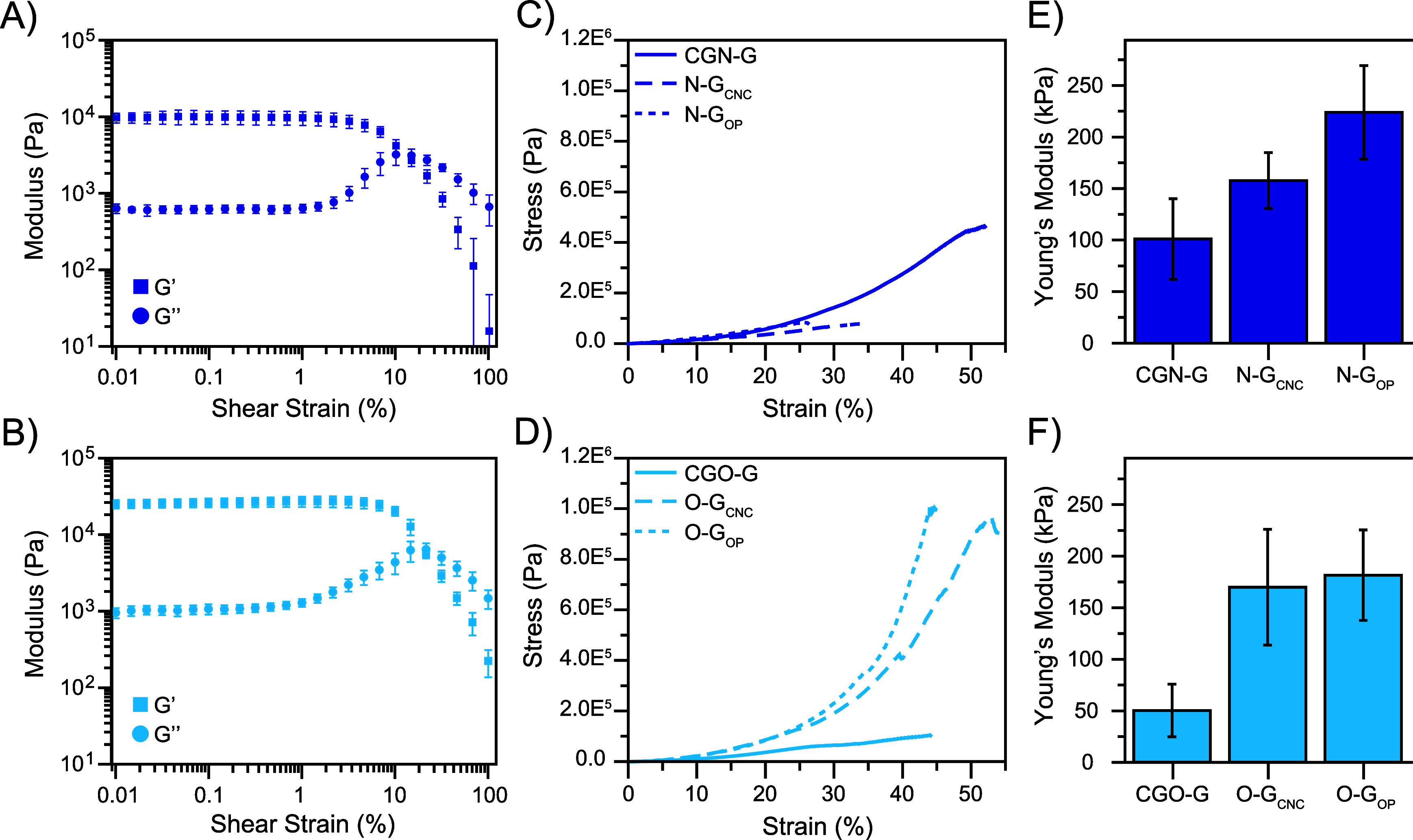
Mechanical characterization of **CGN-G** and **CGO-G** hydrogels. (A, B) Rheological (amplitude sweeps) characterization of (A) **CGN-G** and (B) **CGO-G** (*n* = 5). (C–F) Compression tests of **CGN-G**, **CGO-G**, and control hydrogels **N-G** and **O-G** (*n* = 3). Compression test curves from (C) **CGN-G**, **N-G**_**CNC**_, and **N-G**_**OP**_; and (D) **CGO-G**, **O-G**_**CNC**,_ and **O-G**_**OP**_. Young’s modulus calculated from the initial 0–5% strain in the stress–strain curve of (E) **CGN-G**, **N-G**_**CNC**_, and **N-G**_**OP**_, and (F) **CGO-G**, **O-G**_**CNC**_, and **O-G**_**OP**_.

A summary of the values of the storage moduli determined by rheology for **CGN-G** and **CGO-G**, and control gels **N-G**_**CNC**_ and **O-G**_**CNC**_ (i.e., blends with CNCs), and **N-G**_**OP**_ and **O-G**_**OP**_ (i.e., only cross-linked polymers) (Figure S13) is compiled in Table 1. The data reveal that the *G*′ values of **CGN-G** and **CGO-G** are substantially lower than those of all controls (Table 1). Since higher cross-linking density typically leads to increased *G*′, this suggests that cross-link formation is more effective in the control gels than in the HNP gels. Thus, it appears that, at the low CNC contents employed here, the mechanical properties are dominated by polymer graft entanglement and solvation effects, rather than by cross-linking density and nanoparticle reinforcement. The minor difference in the *G*′ values between **N-G**_**OP**_ and **N-G**_**CNC**_, and between **O-G**_**OP**_ and **O-G**_**CNC**_, further confirms that the CNCs provide a negligible contribution to the overall stiffness of the material due to their low concentration overall (i.e., 0.1–0.2 wt %; see page S6). Plausibly, the loss of crystallinity of the CNCs caused by the synthetic transformations (vide supra) also plays an important role in the poor mechanical reinforcement observed in HNP-based gels. We speculate that a possible way to increase the *G*′ value of **CGN-G** and **CGO-G** could rely on (dramatically) lowering the grafting density and/or the length of the grafts, as this would likely reduce (the influence of) chain entanglement, preserve CNC crystallinity, and consequently enhance the influence of cross-linking.

**Table 1:** Storage Modulus from Amplitude Sweeps of HNP Gels (**CGN-G**, **CGO-G**) and Control Gels (**N-G**, **O-G**), Both as Prepared (DMSO) and Solvent Exchanged (Water)

Storage^a^ (*G*′) and Young’s modulus^b^ (*E*)			
	**CGN-G**	**N-G**_**CNC**_	**N-G**_**OP**_
*G*′—DMSO	15.3 ± 2 kPa	32 ± 8 kPa	33 ± 10 kPa
*G*′—Water	9.8 ± 2 kPa	23 ± 3 kPa	22 ± 4 kPa
*E*—Water	101 ± 39 kPa	158 ± 27 kPa	224 ± 45 kPa

Storage^a^ (*G*′) and Young’s modulus^b^ (*E*)			
	**CGO-G**	**O-G_CNC_**	**O-G_OP_**
*G*′—DMSO	13 ± 3 kPa	39 ± 10 kPa	46 ± 14 kPa
*G*′—Water	26 ± 3 kPa	45 ± 6 kPa	33 ± 14 kPa
*E*—Water	50 ± 26 kPa	170 ± 56 kPa	181 ± 44 kPa

a Determined within the linear viscoelastic region (0.01–0.1%) at 10 rad/s.

b Determined from the slope of the initial 0–5% strain.

To gain further insight into the mechanical properties of the HNP gels and how they compare to their control counterparts, we performed compression tests on all species (Figure 3C–F). All gels were compressed until fracture at a speed of 100 μm s^–1^, and their respective Young’s Modulus (*E*) was calculated from low strains (1–6%). The average Young’s modulus of **CGN-G** was higher than that of **CGO-G** (101 ± 39 kPa vs 50 ± 26 kPa), although it should be noted that the variability leads to overlap within error (Figure 4E,F). The relatively large standard deviations primarily reflect sample-to-sample heterogeneity introduced during solvent exchange from DMSO to water, which can lead to local variations in shrinkage, water content, and effective network density in these soft gels.

**Figure 4:**
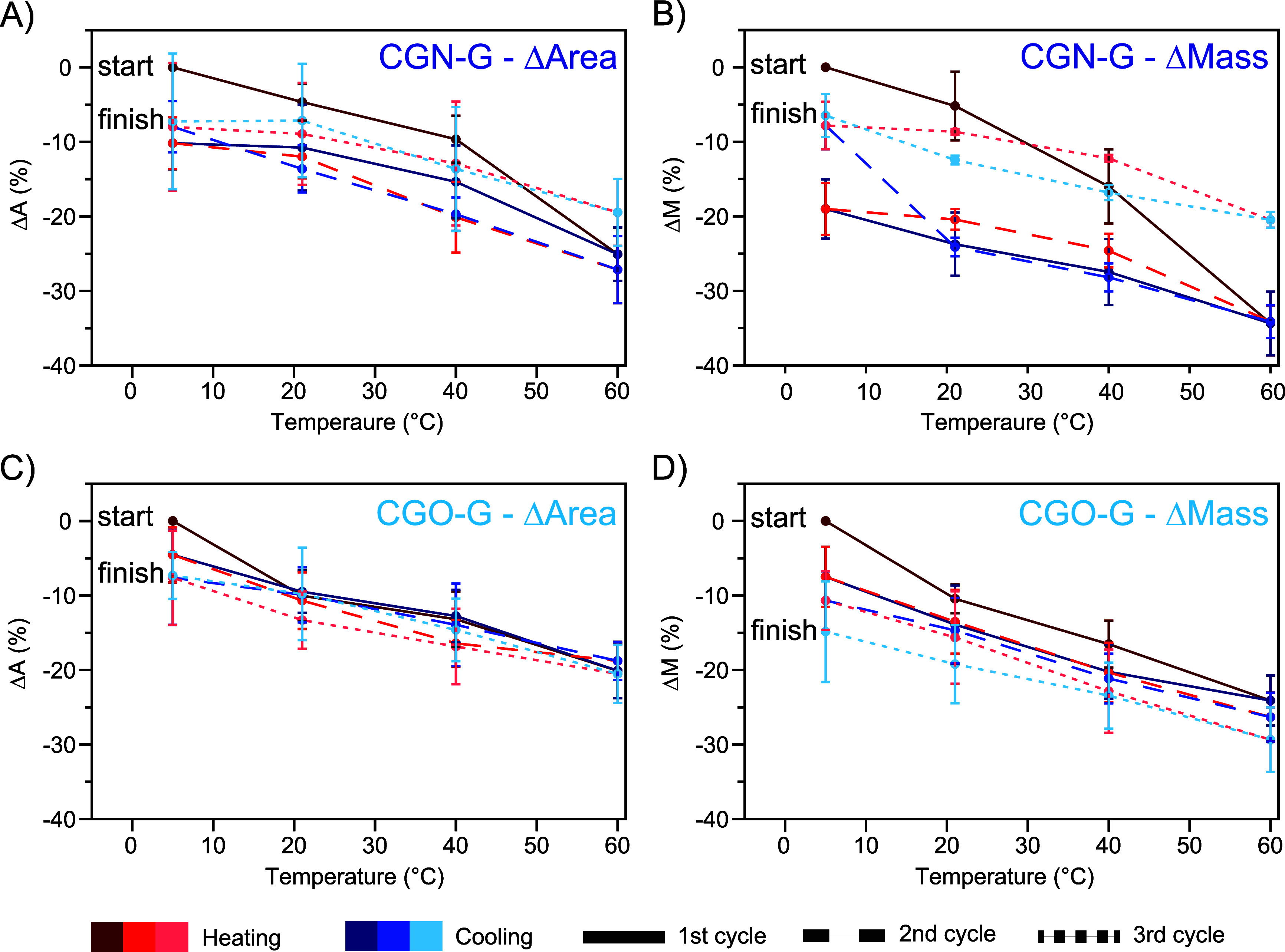
Temperature-dependent deformation of **CGN-G** and **CGO-G** during three consecutive heating (red) and cooling (blue) cycles (1st: solid, 2nd: dashed, 3rd: dotted lines). Deformation was quantified as the relative change in cross-sectional area (Δ*A* = (*A_t_* – *A*_0_)/*A*_0_) and mass (Δ*M* = (*M_t_* – *M*_0_)/*M*_0_), where *A*_0_ and *M*_0_ are the initial area and mass, and *A_t_* and *M_t_* are the values after 10 min at temperature *t*. (A, B) Relative changes in cross-sectional area (A) and mass (B) for **CGN-G**. (C,D) Relative changes in cross-sectional area (C) and mass (D) for **CGO-G**.

The Young’s modulus of the control systems **N-G**_**CNC**_ and **N-G**_**OP**_ was higher than that of **CGN-G**, which is in line with the higher stiffness of these gels determined by rheology. However, the HNP gel could sustain a larger deformation and was significantly tougher than the control gels (Figure 3C). The compression tests carried out on **CGO-G** (and control gels) revealed contrasting behavior compared to **CGN-G**, instead. **CGO-G** could reach the same extent of deformation as **O-G**_**CNC**_ and **O-G**_**OP**_ but did not display the strain stiffening behavior of the control gels (Figure 3D). Overall, these results indicate that polymer chain entanglement likely dominates the mechanical properties of HNP gels, and the distinct responses of **CGN-G** and **CGO-G** demonstrate how variations in polymer composition can lead to markedly different gel properties.

Finally, we investigated the temperature-dependent swelling behavior of **CGN-G**, **CGO-G**, and related control systems (Figures 4A–D and S14–S15). The experiments were performed by immersing the hydrogels in a DI water bath, with each immersion step being accompanied by an increase in temperature. The temperature of the water bath was set at 5 °C, 21 °C (ambient lab temperature), 40 °C, and 60 °C, and the hydrogels were equilibrated for 5 min after reaching the desired temperature prior to measuring the mass and cross-sectional surface area. The temperature steps were subsequently reversed from 60 to 5 °C, passing through 40 and 21 °C, to assess the hydrogels’ ability to reswell upon cooling. This cycle was repeated a total of three times for each hydrogel, with the materials being allowed to re-equilibrate at 5 °C overnight between cycles.

Both **CGN-G** and **CGO-G** showed LCST behavior, as the cross-sectional area and mass (i) decreased as the temperature was raised, and (ii) increased again when the temperature was decreased (Figure 4). The temperature response of **CGN-G** showed a discontinuous decrease in cross-sectional area and mass (Figure 4A,B), while **CGO-G** displayed what appears to be a more linear response (Figure 4C,D). The control gels **N-G**_**CNC**_ and **N-G**_**OP**_ exhibit more pronounced hysteresis and less discontinuous behavior than their HNP counterparts (Figure S14), while **O-G**_**CNC**_ and **O-G**_**OP**_ show similar linear behavior, albeit with less shrinkage (Figure S15). LCST systems typically display discontinuous behaviors.^19,21^ Indeed, measurements based on intensity autocorrelation functions via diffusing wave spectroscopy on control polymers **N** and **O** revealed onset temperatures and LCSTs around 50 and 65 °C, and 30–40 and 56 °C, respectively (Figure S16). We conclude that cross-linking of the HNP and free polymers results in (i) lowering of the onset temperature, and (ii) a significant deviation from discontinuous behavior, most notably for the POEGA-based systems.

Hysteresis is a common characteristic of thermoresponsive polymers and hydrogels being subjected to temperature cycling.^19,87,88^ Previous literature on gels based on PNIPAm established that during the coil-to-globule transition, the PNIPAm chains adopt a number of distinct conformations due to inter- and intrachain hydrogen bonding interactions between amide groups, which can lead to further aggregation.^87,88^ This behavior ultimately limits the rehydration of the chains below the LCST, especially in **CGN-G**, although both **CGN-G** and **CGO-G** exhibit hysteresis and an irreversible decrease in mass and cross-sectional surface area upon temperature cycling (Figure 4). For **CGN-G**, hysteresis was dramatic in the first temperature cycle and reduced significantly in the following cycles, while hysteresis was less pronounced for **CGO-G**, indicating a reduced tendency of the POEGA chains to aggregate further when heated (Figure 4).

The first heating and cooling cycle allowed us to remove most of the irreversible effects, as the subsequent second and third cycles exhibited more stable and reproducible behavior (Figure 4). The relative changes in cross-sectional area and mass of **CGN-G** remained largely similar in the second and third cycles: the cross-sectional area of **CGN-G** showed a shrinkage of −9.9% (second cycle) and −4.9% (third cycle) at 40 °C, and −17.0% (second cycle) and −11.5% (third cycle) at 60 °C (Figure 4A). The trend for the mass mirrors that of the cross-sectional area, showing a relative change of −5.6% (second cycle) and −4.4% (third cycle) at 40 °C, and −15.1% (second cycle) and −12.7% (third cycle) at 60 °C (Figure 4B). A similar behavior was observed for **CGO-G**: the cross-sectional area shrank by −12.4% and −10.0% at 40 °C, and −14.9% and −13.9% at 60 °C during the second and third cycles, respectively (Figure 4C). The mass decreased by −13.8% (second cycle) and −14.0% (third cycle) at 40 °C, and −20.3% (second cycle) and −20.9% (third cycle) at 60 °C, instead (Figure 4D).

When comparing the temperature response of HNP hydrogels and relative controls, one notices minor differences in the LCST interval of the two systems. However, different behaviors appear when considering the degree of shrinking/swelling. Both **N-G**_**OP**_ and **N-G**_**CNC**_ exhibit larger changes in mass (−60%) compared to **CGN-G** (−30%) (Figure S14), whereas **O-G**_**OP**_ and **O-G**_**CNC**_ show minimal changes (−5%) compared to **CGO-G** (−20%) (Figure S15).

Overall, the data confirms that both HNP and control gels exhibit reversible LCST-type behavior, but the amplitude and sharpness of the response depend strongly on polymer architecture. For PNIPAm-based networks, the control **N-G** gel series shows larger swelling/deswelling amplitudes than the corresponding HNP gels, indicating that the dense grafting and entanglement in the latter restrict the full collapse and rehydration of the chains. Conversely, the POEGA-based HNP gels display a more pronounced and reproducible response than their control analogues, suggesting that the specific solvation and mobility of the POEGA grafts allow a more cooperative network contraction. Overall, these results show that the actuation behavior can be modulated by graft chemistry and architecture, and that the CNC-grafted design primarily serves to define network connectivity rather than to provide mechanical reinforcement. These results define clear design constraints for covalently cross-linked HNP-based gels and highlight the trade-offs associated with dense grafting and solvation effects.

## Conclusion

In summary, polymer-grafted cellulose nanocrystals can serve as building blocks for covalently cross-linked thermoresponsive hydrogels. Using surface-initiated ATRP, we grafted PNIPAm- and POEGA-based gradient copolymers bearing terminal allyl groups from CNCs and cross-linked the resulting hairy nanoparticles through UV-mediated thiol–ene chemistry. The gels form rapidly and exhibit reversible temperature-dependent swelling, confirming the preparation of a continuous, stimuli-responsive network.

Rheological and mechanical measurements revealed that these gels possess moduli in the tens of kPa and different solvation-dependent behaviors: PNIPAm-based networks soften upon hydration, while POEGA-based systems stiffen. This result highlights the impact of the chemical nature of the grafts on the macroscopic mechanics of the materials. However, we also found that a 0.1–0.2 wt % CNCs content in the final materials offers a negligible reinforcing effect, suggesting that polymer chain entanglement and cross-link density primarily dictate response and properties at these low CNC concentrations. Future work will address this limitation by increasing the CNC content through reduced grafting density and chain length. Moreover, we deliberately investigated formulations comprising only one type of HNP, but the modularity of our approach should allow us to combine multiple HNPs into a single gel, potentially unlocking more complex (thermoresponsive) behavior in the future.

Our results also showed that the present hairy-nanoparticle gel formulations do not yet outperform conventional “two-component” polymer–CNC composites in absolute mechanical terms. Nevertheless, our work has established the synthetic feasibility and inherent limitations of a covalently cross-linked “one-component” nanocomposite architecture in which CNCs are integral elements of the polymer network rather than passive fillers. While solid, non-cross-linked “one-component” HNP nanocomposites have been reported to exhibit markedly different mechanical behavior compared to their two-component counterparts,^55,57^ the same enhancement has not been observed in our gels. This contrast highlights how dense graft entanglements and bottlebrush-like behavior can stiffen dry HNP-based solids, but such an effect is not sustained under the highly solvated conditions typical of hydrogels.

Overall, this work clarifies how graft chemistry, network architecture, and solvation collectively govern the thermoresponsive and mechanical behavior of HNP-based hydrogels at low CNC contents. These findings provide an experimental baseline for future optimization, particularly by increasing CNC incorporation through tuning graft length and density. Finally, although our primary focus was on material design and feasibility, we note that all hydrogel components have been used previously in biomedical contexts. As such, future studies on biocompatibility will be important for evaluating the suitability of these materials for biomedical applications.

## Supplementary Material



## Notes

Experimental details, materials, methods, calculations, ^1^H NMR spectra of polymerization time points and control species, TEM and AFM images, titration data, additional TGA and SEC data of polymer-grafted CNCs and control polymers, additional rheological and temperature responsiveness data of gels, diffusing-wave spectrograms of polymers (PDF)

## Abbreviations

^1^H NMR: proton nuclear magnetic resonance
AFM: atomic force microscopy
AGET: activator-generated electron transfer
AMA: allyl methacrylate
ATR-FTIR: attenuated total reflection Fourier transmission infrared spectroscopy
ATRP: atom-transfer radical polymerization
BiBB: bromoisobutyryl bromide
CGN: CNC-*g*-P­(NIPAm-*grad*-NHEAm)
CGN-A: CNC-*g*-P­(NIPAm)-*grad*-P­(NHEAm-*stat*-allylacrylamide)
CGN-G: gels of cross-linked CGN-A
CGO: CNC-*g*-P­(HPA-*stat*-HEA)
CGO-A: CNC-*g*-P­(HPA-*stat*-HEA)-*grad*-P­(AMA)
CGO-G: gels of cross-linked CGO-A
CNC: cellulose nanocrystals
CNC-Br: CNCs functionalized with ATRP initiators
DMSO: dimethyl sulfoxide
DI H_2_O: deionized water
DMAP: 4-(dimethylamino)­pyridine
DMF: dimethylformamide
DWS: diffusing wave spectroscopy
E: Young’s modulus
EA: elemental analysis
EDC·Cl: *N*-(3-(dimethylamino)­propyl)-*N*′-ethylcarbodiimide hydrochloride
EtBiB: ethyl bromoisobutyrate
EtOH: ethanol
FTIR: Fourier transmission infrared spectroscopy
*G*′: storage modulus
*G*″: loss modulus
HEA: hydroxyethyl acrylate
HNP: hairy nanoparticles
HOBiB: 2-hydroxyethyl 2-bromoisobutyrate
HPA: hydroxypropyl acrylate
Irgacure 2959: 2-hydroxy-4′-(2-hydroxyethoxy)-2-methylpropiophenone
LCST: lower critical solution temperature
Me_6_TREN: tris­[2-(dimethylamino)­ethyl]­amine
MeOH: methanol
MQ H_2_O: milli-Q water
*M_n_*: number-average molecular weight
MWCO: membrane molecular weight cut-off
N_2_: nitrogen (g)
N: P­(NIPAm-*stat*-NHEAm)
N-A: P­(NIPAm-*stat*-(NHEAm-*stat*-allylacrylamide))
N-G_CNC_: gels of cross-linked N-A with CNCs
N-G_OP_: gels of cross-linked N-A with only the polymer
NHEAm: *N*-(2-hydroxyethyl)­acrylamide
NIPAm: *N*-isopropylacrylamide
O: P­(HPA-*stat*-HEA)
O-A: P­((HPA-*stat*-allylacrylate)-*stat*-(HEA-*stat*-allylacrylate))
O-G_CNC_: gels of cross-linked O-A with CNCs
O-G_OP_: gels of cross-linked O-A with only the polymer
PNIPAm: poly­(*N*-isopropylacrylamide)
POEGA: poly­(oligoethylene glycol acrylates)
PMDETA: *N*,*N*,*N*′,*N*″,*N*″-pentamethyldiethylenetriamine
PTM: pentaerythritol tetrakis­(3-mercaptopropionate)
RAFT: reversible addition–fragmentation chain-transfer
RBF: round-bottom-flask
*R*_*p*_: rate of polymerization
SEC: size exclusion chromatography
SI-AGET: surface-initiated activator-generated electron transfer
SI-ATRP: surface-initiated atom-transfer radical polymerization
SI-CRP: controlled radical polymerization
SSA: specific surface area
TEA: triethylamine
TEM: transmission electron microscopy
TGA: thermogravimetric analysis
THF: tetrahydrofuran
