# Supplementary material for: Thermoresponsive Gels Based on Cross-Linked Polymer-Grafted Cellulose Nanocrystals
Source: Biomacromolecules. 2026 Mar 20;27(4):2555–71. doi: 10.1021/acs.biomac.5c02262 (PMC13081003; doi:10.1021/acs.biomac.5c02262)
Supplement: Supplementary file 1 [file bm5c02262_si_001.pdf]

Supplementary Information to

Thermoresponsive Gels based on Crosslinked Polymer-Grafted Cellulose Nanocrystals

Matilde Folkesson,[a‡] *Justus Paul Wesseler,*[a‡] *Carolina Pierucci,*[a,b] *Chris Rader,*[c] *Christoph Weder,*[a,b] *Alessandro Ianiro,*[a,b,d] *José Augusto Berrocal*[a,e,f*]

*To whom correspondence should be addressed: jberrocal@iciq.es

‡ Equal contribution from these authors

[a] Adolphe Merkle Institute, Chemin des Verdiers 4, 1700 Fribourg, Switzerland.

[b] National Center for Competence in Research (NCCR) Bio-inspired Materials, University of Fribourg, Chemin des Verdiers 4, 1700 Fribourg, Switzerland

[c] Laboratory for Biomimetic Membranes and Textiles, Empa, Swiss Federal Laboratories for Materials Science and Technology, Lerchenfeldstrasse 5, 9014 St. Gallen, Switzerland.

[d] Dep. of Chemistry, Catholic University of Leuven, Celestijnenlaan 200F, 3001 Leuven, Belgium.

[e] Institute of Chemical Research of Catalonia (ICIQ), Barcelona Institute of Science and Technology (BIST), Avda. Països Catalans 16, 43007 Tarragona, Spain.

[f] Catalan Institution for Research and Advanced Studies (ICREA), Pg. Lluís Companys 23, 08010 Barcelona (Spain)

Table of Contents

Characterization of Non-grafted Polymers 2
Calculations 2
Supporting Figures 6
References 20

Characterization of Non-grafted Polymers

The composition of **N** was determined by ^{1}H NMR and SEC of the purified product. In the ^{1}H NMR spectra of **N** (**Figure S7.A**), the characteristic signal of -O*H* for NHEAm at 4.90 ppm (proton **b**, *I* = 0.54, H = 1) and the characteristic signal of -C*H*(CH_3)$_2$ for NIPAm at 3.83 ppm (proton **c**, *I* = 1.00, H =1) were compared to yield a 65:35 composition of NIPAm / NHEAm. The M_n (18,700) and *Đ* (1.20) of the polymer were determined by SEC (**Figure S9.A**).

The composition of **O** was determined by ^{1}H NMR and SEC of the purified product. From the ^{1}H NMR spectra (**Figure S8.A**) of **O** a similar degree of polymerization of HPA and HEA can be determined by comparing characteristic peaks of their side chains, **m** at 1.08 ppm (3.05, H = 3) and **h** at 3.55 ppm (2.00, H = 2), respectively. The M_n (23040 g/mol) and *Đ* (1.23) of the polymer were determined by SEC (**Figure S9.C**). In the SEC traces, a small shoulder can be observed, indicating that some termination via combination took place.

Calculations

Grafting Density Calculation. CNC-Br dimensions (w = 11 nm, h= 6 nm) were determined by TEM and AFM. To calculate their specific surface area (SSA) in bulk, the CNCs are assumed to be infinitely long cuboids, making the area of the two rectangular ends negligible.[S1] The mantle area of the segment, excluding the area of the sides, was calculated by **Eq.1**:

$$A_{Seg} = 2 \times (w + h) \times l \qquad \textbf{Eq. 1}$$

The volume of a cuboid segment with a specific length (l = x nm) was calculated by **Eq.2**.

$$V_{Seg} = w \times h \times l \qquad \textbf{Eq. 2}$$

The SSA was then calculated using the density of crystalline cellulose (1.58 g/mL) according to **Eq.3**:

$$SSA = \frac{A_{Seg}}{\rho_{Cellulose} \times V_{Seg}} = \frac{2(w+h)\times l}{\rho_{Cellulose}\times w \times h \times l} = \frac{2\times(w+h)}{\rho_{Cellulose}\times w \times h} \qquad \textbf{Eq. 3}$$

The grafting density of initiators (σ_i) on the CNCs was calculated by **Eq.4**[S2]:

$$\sigma_i = \frac{\frac{w_i}{Mw_i} \times N_A}{(1-w_i) \times SSA \times 10^{18}} \qquad \textbf{Eq. 4}$$

Where w_i ($w_i = (w_{Br} / M_{Br}) * Mw_i$) is the weight fraction of initiators (Mw_i = 150 g/mol) determined by elemental analysis of Br (w_{Br} = 0.2757 g Br / g CNC, M_{Br} = 79.9 g/mol) and N_A is Avogadro's constant. Using **Eqs.1-4** the **CNC-Br** has an SSA of 341 m^2 / g and a σ_i of ~12.6 nm^{-2}.

Determination of Theoretical Polymer *M_n* and Degree of Polymerization. The theoretical number average molecular weight (*Mn*) of polymer chains was calculated according to **Eq.5**.

$$DP = \frac{[M]_0}{[I]_0} \times \rho \qquad \textbf{Eq.5}$$

Where DP is the degree of polymerization, $[M]_0, [I]_0$ the initial monomer and initiator concentration respectively and ρ the conversion. Multiplying the DP by the molecular weight of the respective monomer or comonomers then yielded the theoretical M_n.

Determination of Allyl Groups in CGN-A and CGO-A. The allyl-functionalization of the sidechains of the polymer-grafted CNCs was determined by ^{1}H NMR.

For **CGN-A**, the allyl content was determined by comparing the integral of the peak corresponding to the ^{1}H situated at the allyl groups unsaturated carbon at 5.80 ppm (**Figure 1.A**, **CGN-A**, proton **a**) to the integral of the amide peak at 7.43 ppm (**Figure 1.A**, **CGN**-**A**, proton **A**), which accounts for all acrylamide species. The molar percentage of allyl-functional units was calculated as follows by **Eq.6**:

$$\varphi_{Allyl} = \frac{Int_{Allyl}}{Int_{Amide}} \times 100 \qquad \textbf{Eq. 6}$$

The remaining NHEAm content was then determined by subtracting the amount of allyl units from the initial amount of NHEAm units in **CGN** (**Eq.7**), due to the overlapping peaks of the NHEAm hydroxyl and the CH_2=CH- of the allyl-functional acrylamides (**Figure 1.A**, **CGN-A**, protons **b** & **c**)

$$\varphi_{NHEAm(CGN-A)} = \varphi_{NHEAm(CGN-OH)} - \varphi_{Allyl} \qquad \textbf{Eq. 7}$$

Due to the high initiator grafting density and polymer molecular weight, the mass contribution of the CNCs in the final polymer-grafted CNCs was deemed negligible. Calculations to determine the molar amount of allyl units and appropriate amount of PTM to add were calculated according to **Eq.8** and **Eq.9**:

$$n_{Allyl} = \frac{m_{polymer}}{\overline{Mw}_{RU}} \times \varphi_{Allyl} \qquad \textbf{Eq. 8}$$

$$\overline{Mw}_{RU} = \sum_{i=1}^{n} Mw_i \, \varphi_i \qquad \textbf{Eq. 9}$$

Where $\overline{Mw}_{RU}$ is the average molecular weight of the polymer repeat unit accounting for the molar fractions of NIPAm/NHEAm/AllylAm residues in the polymer.

For **CGO-A**, the allyl content was determined by averaging two methods:

i) calculating the degree of polymerization of AMA (**Figure S6**) by comparing the integral of the aliphatic proton peak at 4.64 ppm (**d_m)** in AMA to the polymer peak at 4.46 ppm (**d_p**) calculated as follows by **Eq.10**:

$$\varphi_{Allyl} = \frac{Int_{p(Allyl)}}{Int_{p(Allyl)} + Int_{m(Allyl)}} \times 100 \qquad \textbf{Eq. 10}$$

And ii) comparing the integrals in the purified sample (**Figure 1.A, CGO-A**) of the allyl group's unsaturated and saturated carbons at 5.91 ppm (**a**) and 5.30 ppm (**b**), to the integral of the methyl group of polymerized HPA at 1.06 ppm (**m**) calculated as follows by **Eq.11**:

$$\varphi_{Allyl} = \frac{Int_{p(Allyl)}}{Int_{p(methyl)}} \times 100 \qquad \textbf{Eq. 11}$$

The final value used was the average of the two.

Determination of Allyl Groups in N-A and O-A. The allyl-functionalization of the side chains of the free polymers **N-A** and **O-A** was determined by ^{1}H NMR.

For **N-A**, the peak **b** at 5.81 ppm (I = 0.19, H = 1), which is characteristic of the pendant allyl groups, was compared to peak **a** at 7.29 ppm (I = 1.44, H = 1) assigned to the N-H proton of all acrylamide species in the polymer **(Figure S7.B)**. The degree of modification was calculated to be 13.2 % according to **Eq.12**:

$$\varphi_{Allyl} = \frac{Int_{Allyl}}{Int_{Acrylamide}} \times 100 \qquad \textbf{Eq. 12}$$

Finally, a polymer composition of [NIPAm]:[NHEAm]:[AllylAcrylamide] 65 .0 : 21.8 : 13.2 was determined for **N-A**.

For **O-A**, the allyl content was determined by comparing the average of the integral of peak **a** at 5.81 ppm (I = 1, H = 1) and **b** at 5.01 ppm (I = 2.39, H = 2), characteristic of pendant allyl groups, to the sum of the integral of peaks **h** at 3.55 ppm (I = 6.62, H = 2) and **m** at 1.11 ppm (I = 11.53, H = 3), corresponding to the aliphatic protons in HEA and the methyl group in HPA, respectively

(**Figure S8.B**). Assuming equal functionalization likelihood for HEA and HPA, the degree of modification was calculated as 15.0% using **Eq.13**:

$$\varphi_{Allyl} = \frac{Int_{Allyl}}{Int_{HPA}+Int_{HEA}} \times 100 \qquad \textbf{Eq. 13}$$

Finally, a polymer composition of [HPA]:[HEA]:[AllylAcrylate] 42.5 : 42.5 : 15.0 was determined for **O-A**.

Determination of CNC Content in CGN-G and CGO-G. Since the **CNC-Br** were highly functionalized, only 48 % of their mass is attributed to the CNCs according to **Eq.14**:

$$1 - \frac{\mathrm{m}_{initiator}}{g_{CNC}} = 1 - \frac{0.2756\ \mathrm{g}_{Br}/\mathrm{g}_{CNC} \times 150\ \mathrm{g/mol}_{initiator}}{79.9\ \mathrm{g/mol}_{\ Br}} = 0.48\ \mathrm{g} \qquad \textbf{Eq. 14}$$

The fraction of CNCs in **CGN-A** was estimated to be 0.97 % according to **Eq.15**:

$$\frac{0.201\ g_{CNC-Br} \times 48\ \%}{9.6\ g_{CGN-A\ final\ product}} = 0.97\ \% \qquad \textbf{Eq. 15}$$

While the fraction of CNCs in **CGO-A** was estimated to be 0.7 % according to **Eq.15**:

$$\frac{0.066\ g_{CNC-Br} \times 48\ \%}{4.7\ g_{CGO-A\ final\ product}} = 0.70\ \%$$

Thus, in the dispersion used to prepare the gels, which contain 18 %w/v, **CGO-G** or **CGN-G**, the concentration of CNCs is calculated to be between 0.12 and 0.17 %w/v.

Supporting Figures

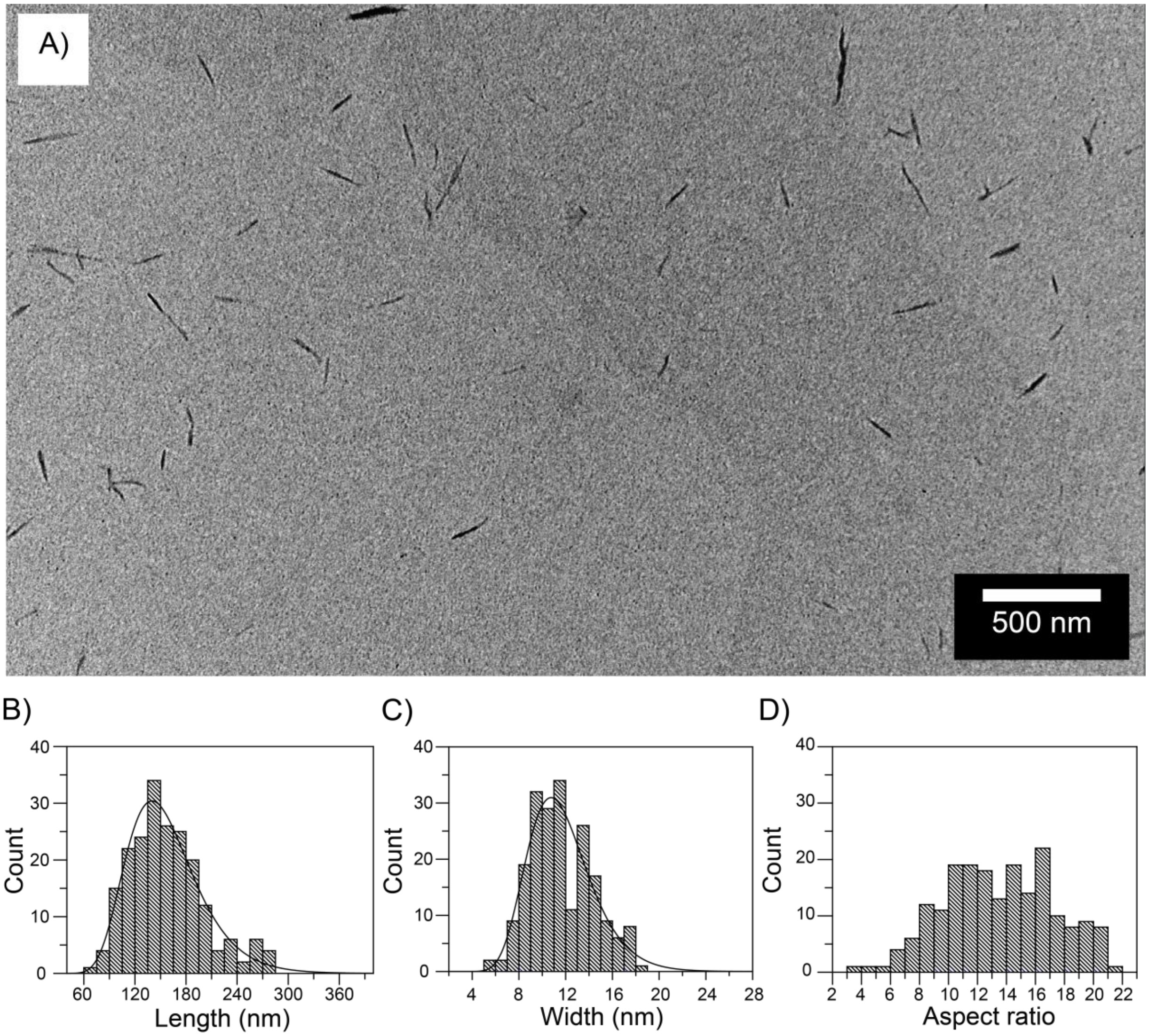

Figure S1. (A) Representative TEM image of pristine CNCs and histograms that show the (B) length, (C) width, and (D) aspect ratio of CNCs measured from 6 TEM images (n = 205)

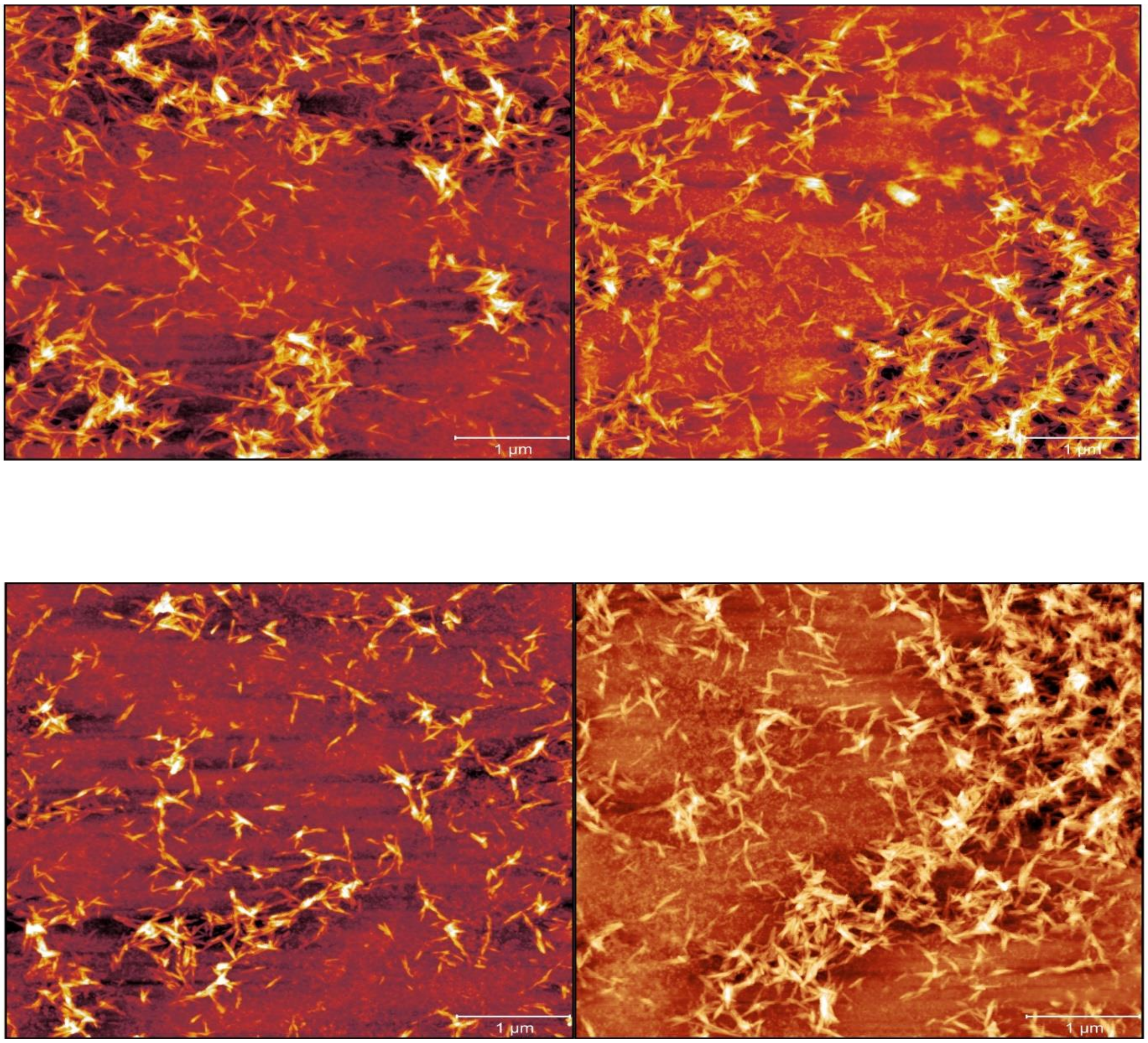

Figure S2. AFM images of CNCs. An average height of 6 ± 1 nm was calculated from 4 AFM images (n = 66)

.

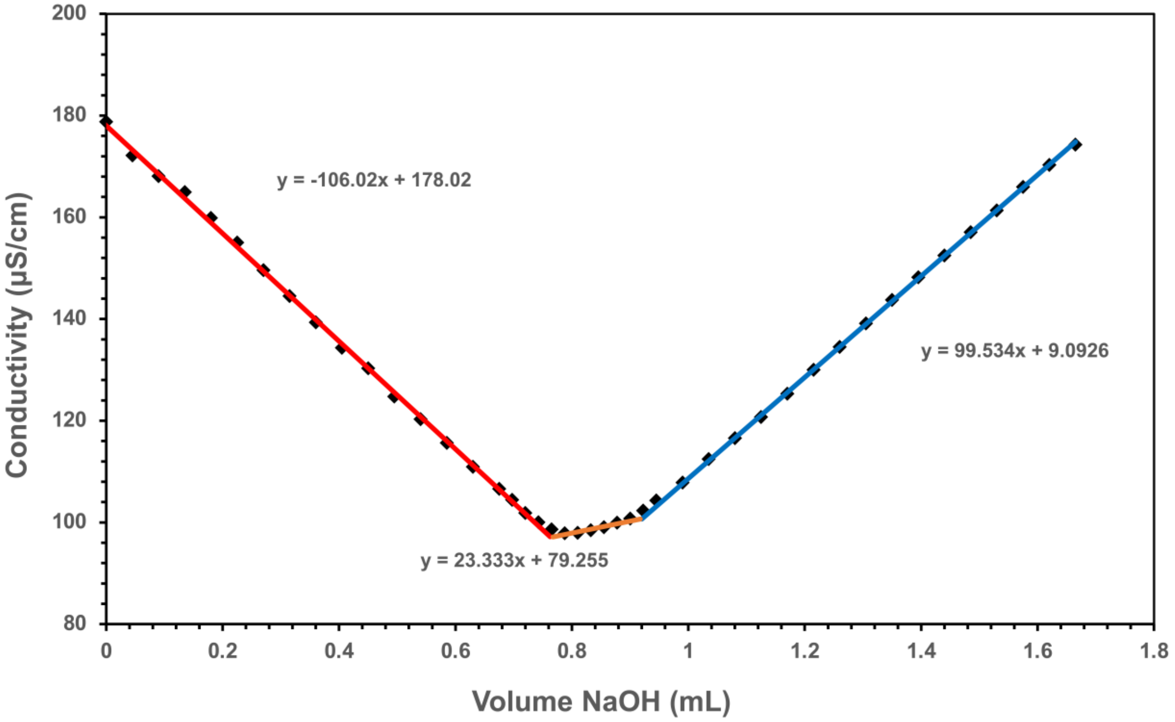

Figure S3. Conductometric titration profile for CNCs against a 0.01 M NaOH solution. Titrations were run in triplicate, and results show a R-OSO_3H concentration of 190 ± 1 mmol kg^{-1}.

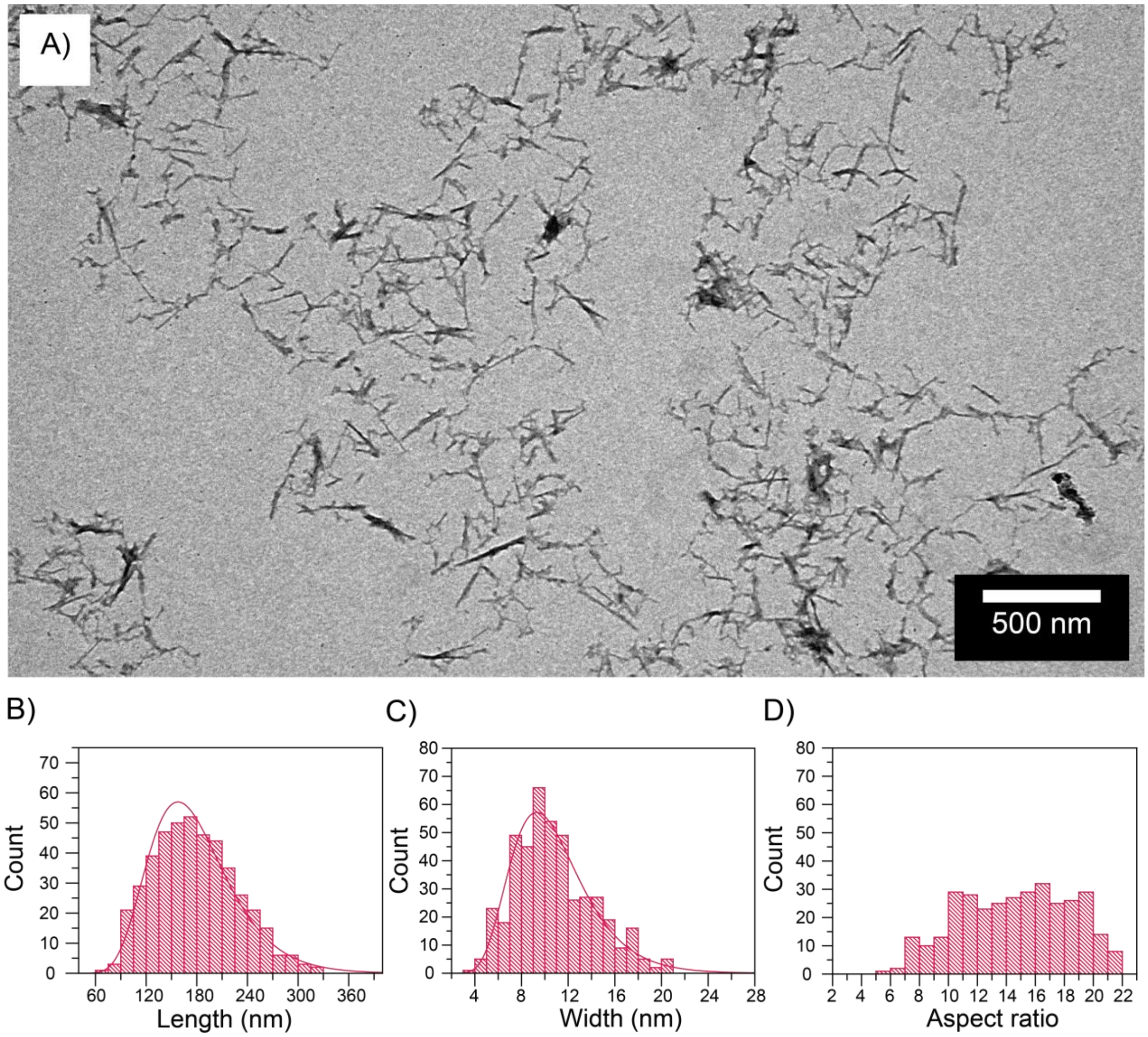

Figure S4. (A) Representative TEM image of **CNC-Br** and histograms that show the (B) length, (C) width, and (D) aspect ratio of **CNC-Br** measured from 11 TEM images (n = 446)

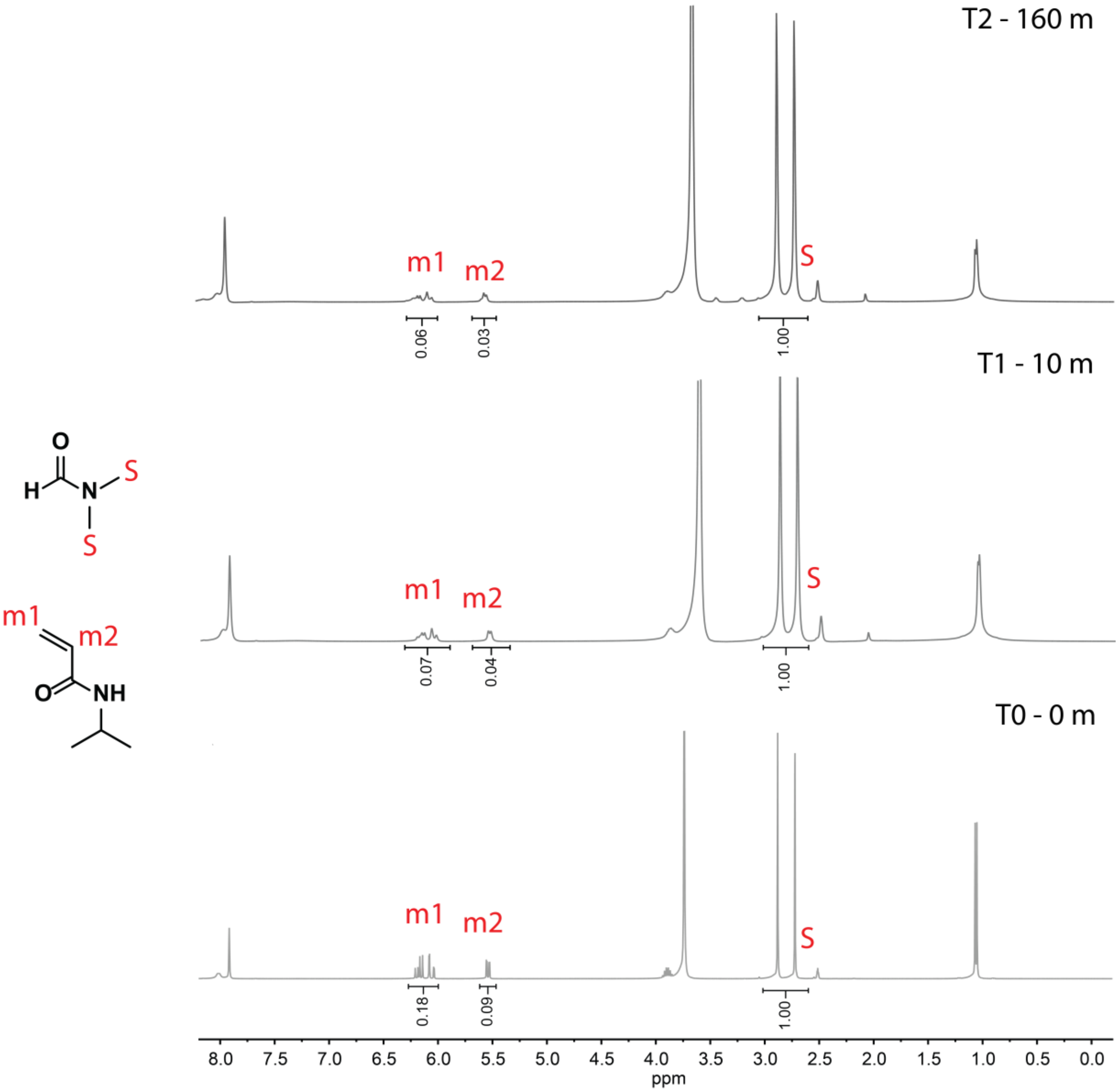

Figure S5. ^{1}H NMR (400 MHz, DMSO-d_6) spectra of time points for the SI-ATRP of NIPAm from **CNC-Br**. The -CH_3 signals of DMF (S) were used as an internal standard to compare against the unsaturated proton signals of NIPAm (m1, m2). By comparing the integral ratio of these signals at a given time point with the integral ratio at T0, it was possible to determine the monomer conversion, and thus the theoretical M_n assuming perfect control over dispersity.

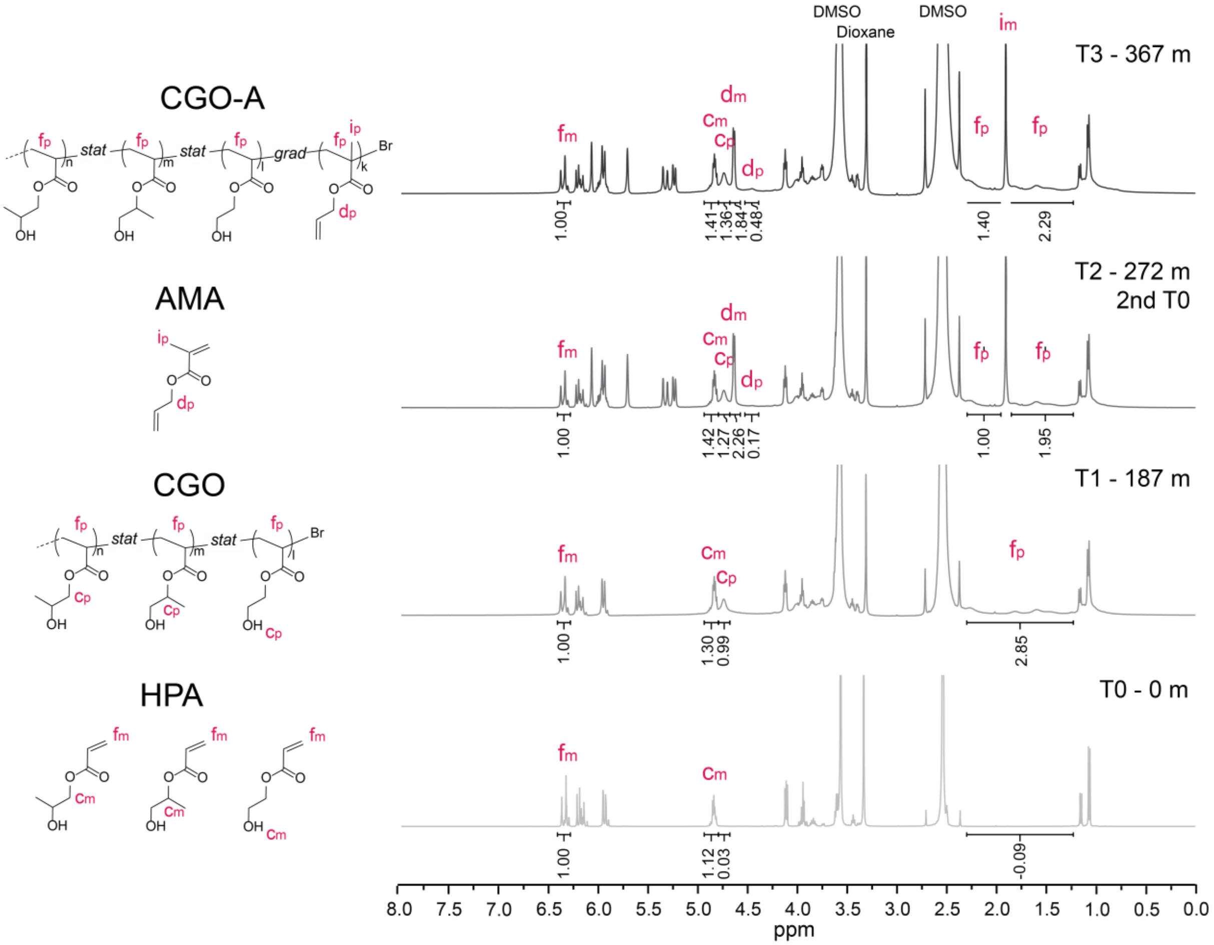

Figure S6. ^1H NMR (400 MHz, DMSO-d_6) time points of the one-pot reaction of **CGO-A** with **CNC-Br** as initiator. First, a statistical co-polymerization of HPA and HEA is carried out (**CGO**), before AMA is added (272 min, 2nd T0) to produce a gradient with an increasing concentration of allyl groups towards the end of the polymer grafts (**CGO-A**).

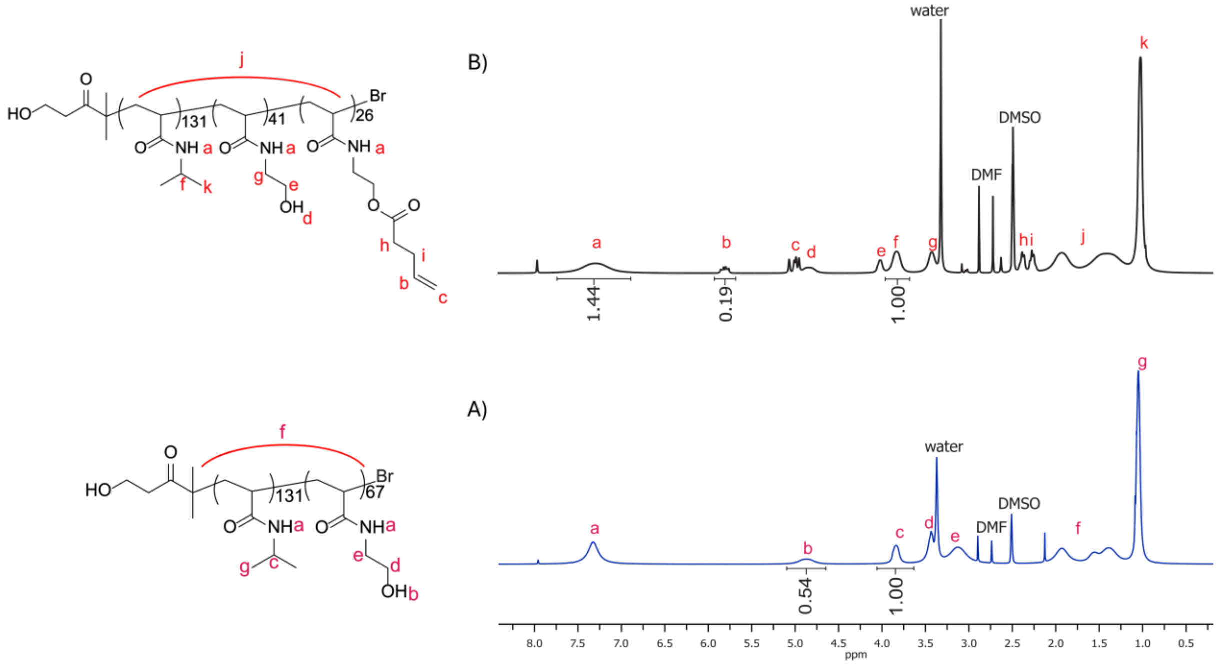

Figure S7. ^{1}H NMR spectra (400 MHz, DMSO-d_6) of non-grafted polymers. (A) P(NIPAm-*stat*-NHEAm) (**N**) and (B) after functionalization with allyl groups (**N-A**).

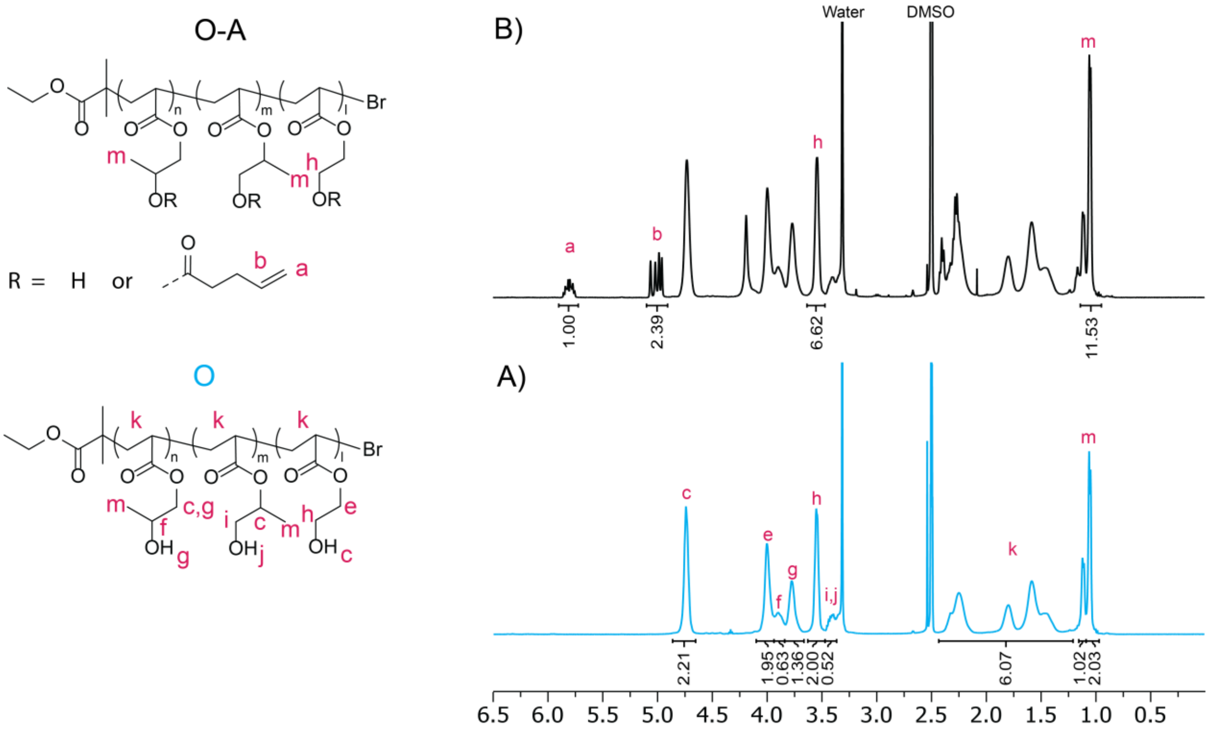

Figure S8. ^{1}H NMR spectra (400 MHz, DMSO-d_6) of non-grafted polymers (A) P(HPA-*stat*-HEA) (**O**) and (B) after functionalization with allyl groups (**O-A**).

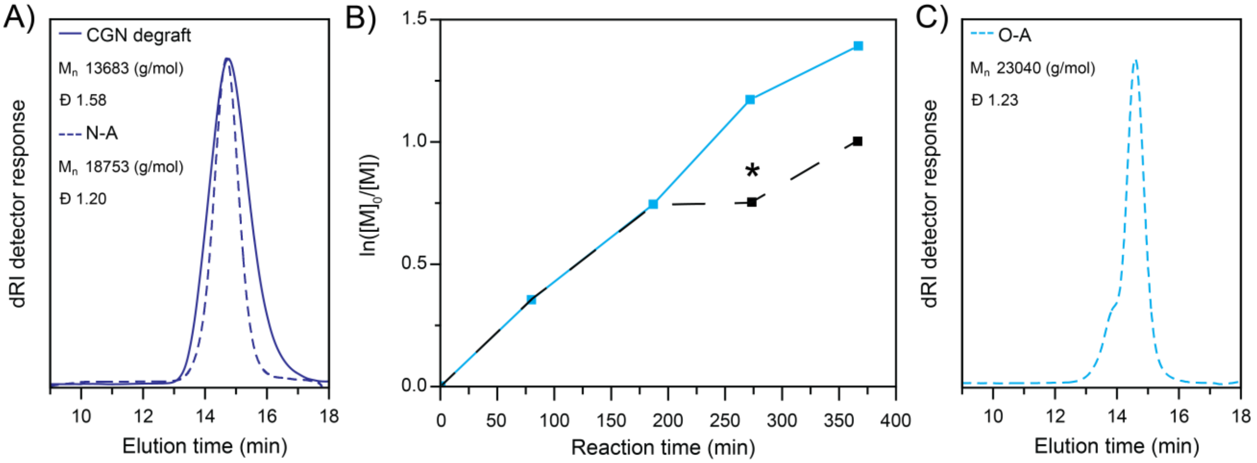

Figure S9. (A) SEC traces of P(NIPAm)-*grad*-P(NHEAm) degrafted from the CNC surface of **CGN** after saponification with 2 % NaOH solution (solid line), and a free model polymer **N-A** (dashed line), displaying the difference in dispersity between SI-AGET-ATRP and AGET-ATRP under similar conditions. (B) Semilogarithmic kinetic plots of monomer conversion vs. time for the SI-ATRP reaction yielding **CGO-A**. Data points for the conversion were calculated from integration of the ^{1}H NMR traces of aliquots taken at set time points. Black line: change in monomer concentration of HPA and HEA (72.3 mmol) after the addition of AMA (22.3 mmol) at 272 min (*). Blue line: change in monomer concentration of HPA and HEA in the **CGO-A** system (omitting AMA). (C) SEC trace of model polymer **O-A**.

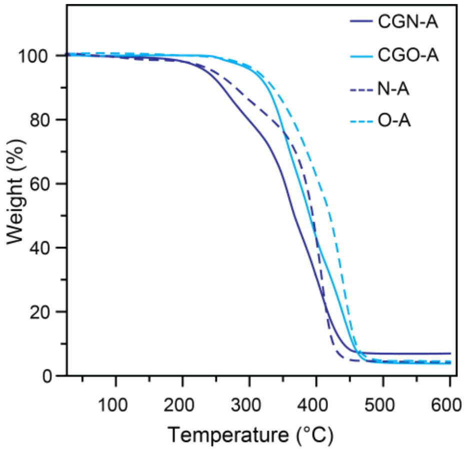

Figure S10. TGA traces of **CGN-A**, **CGO-A**, and control **N-A** and **O-A**.

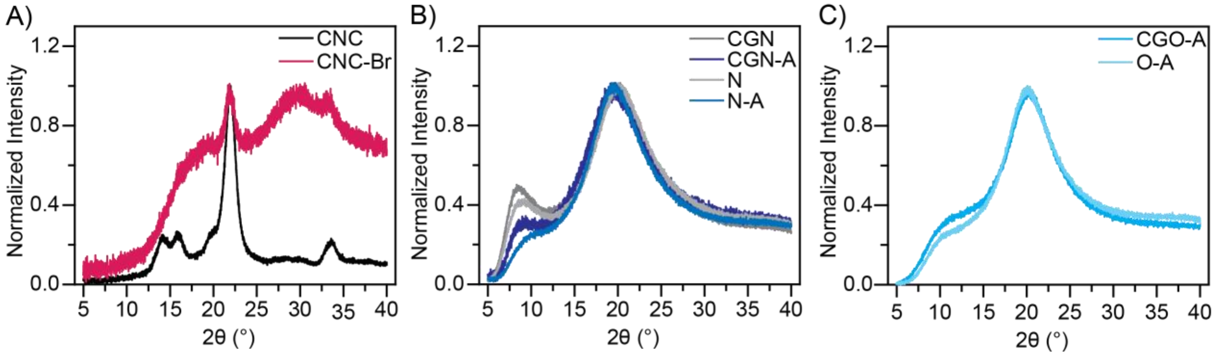

Figure S11. X-ray diffraction (XRD) patterns of (A) pristine CNCs and **CNC-Br**, (B) **CGN**, **CGN-A**, and corresponding model polymers **N** and **N-A**, and (C) **CGO-A** and corresponding model polymer **O-A**.

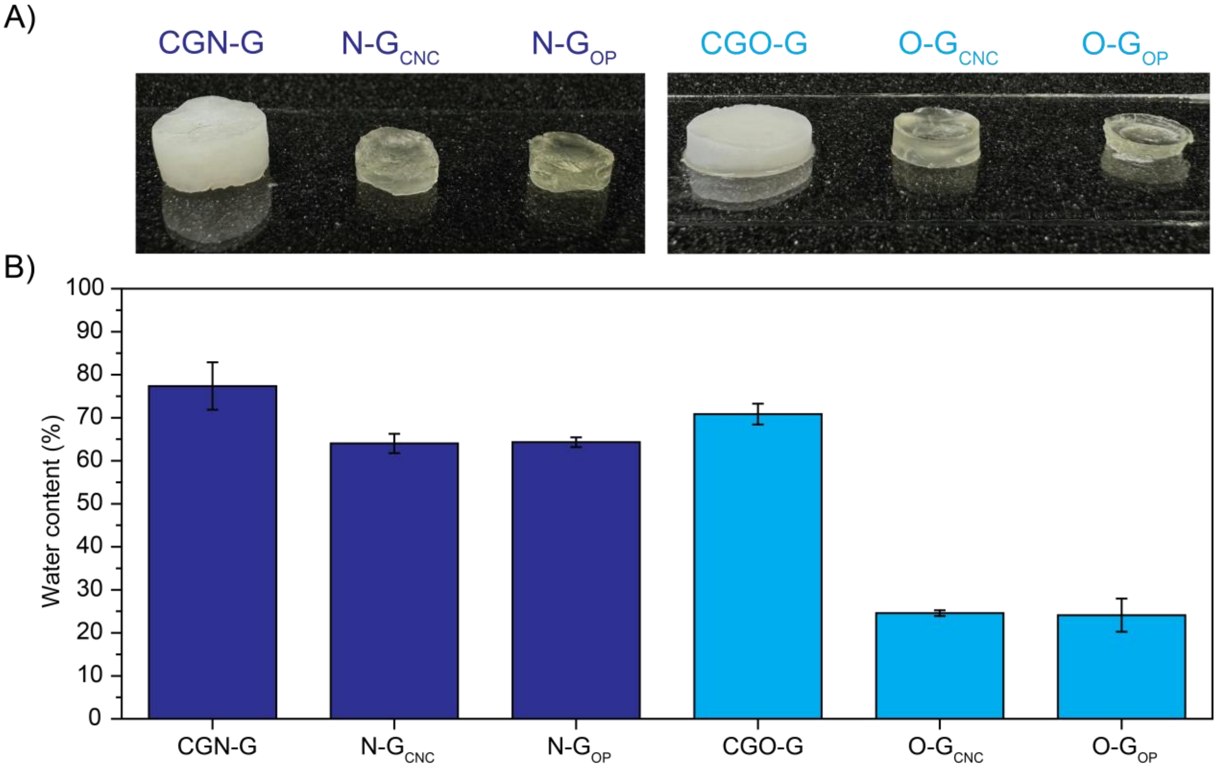

Figure S12. (A) Photographs of **CGN-G**, **N-G**$_{\mathbf{CNC}}$, and **N-G**$_{\mathbf{OP}}$; **CGO-G**, **O-G**$_{\mathbf{CNC}}$, and **O-G**$_{\mathbf{OP}}$ after solvent exchange with water. (B) Water content in gels **CGN-G**, **N-G**, **CGO-G**, and **O-G** determined from the drying experiments.

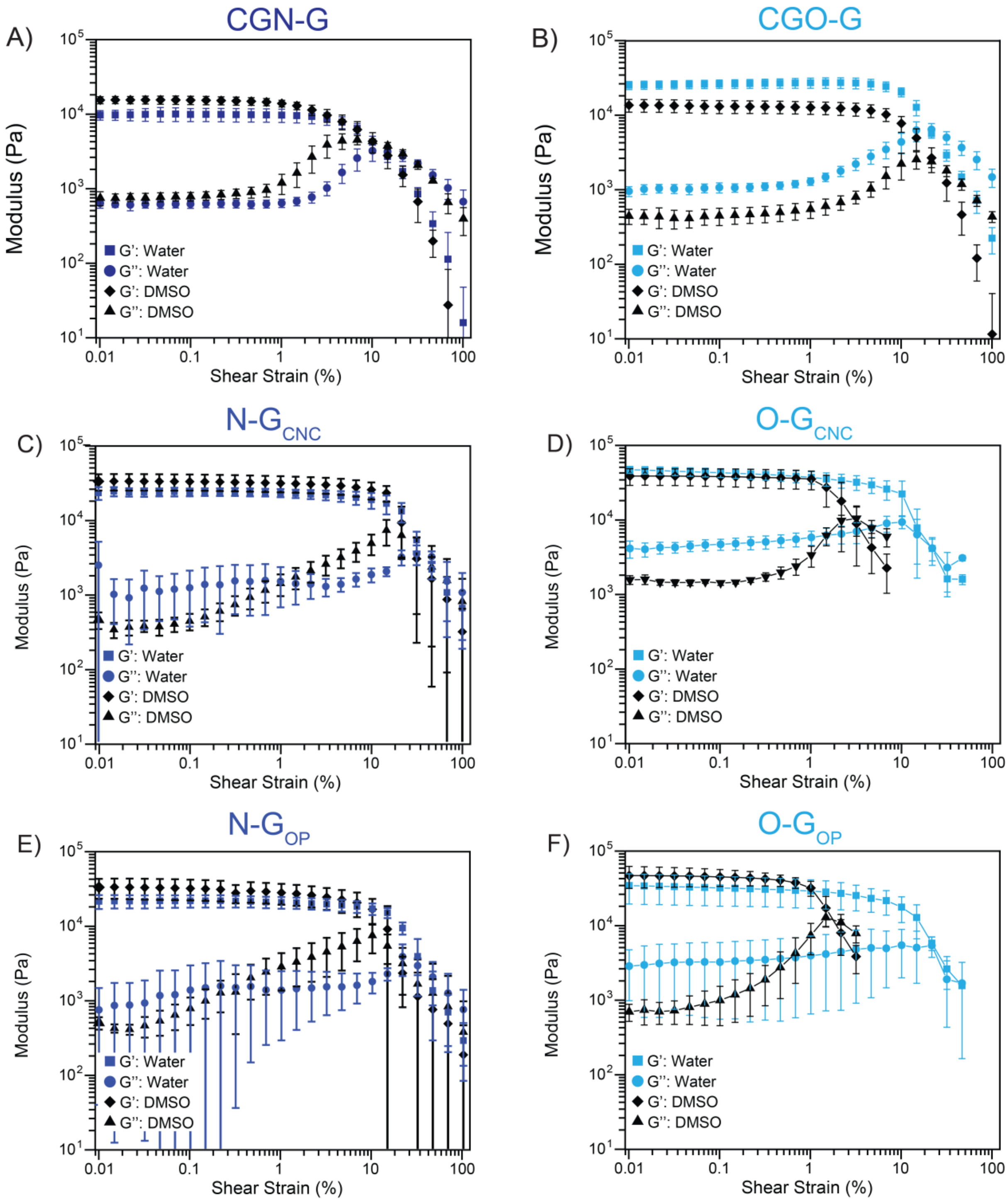

Figure S13. Amplitude sweeps of gels "as prepared" organogels and after solvent exchanged to DI H_2O (A) **CGN-G**, (B) **CGO-G**, (C) **N-G$_{CNC}$**, (D) **O-G$_{CNC}$**, (E) **N-G$_{OP}$**, and (F) **O-G$_{OP}$**.

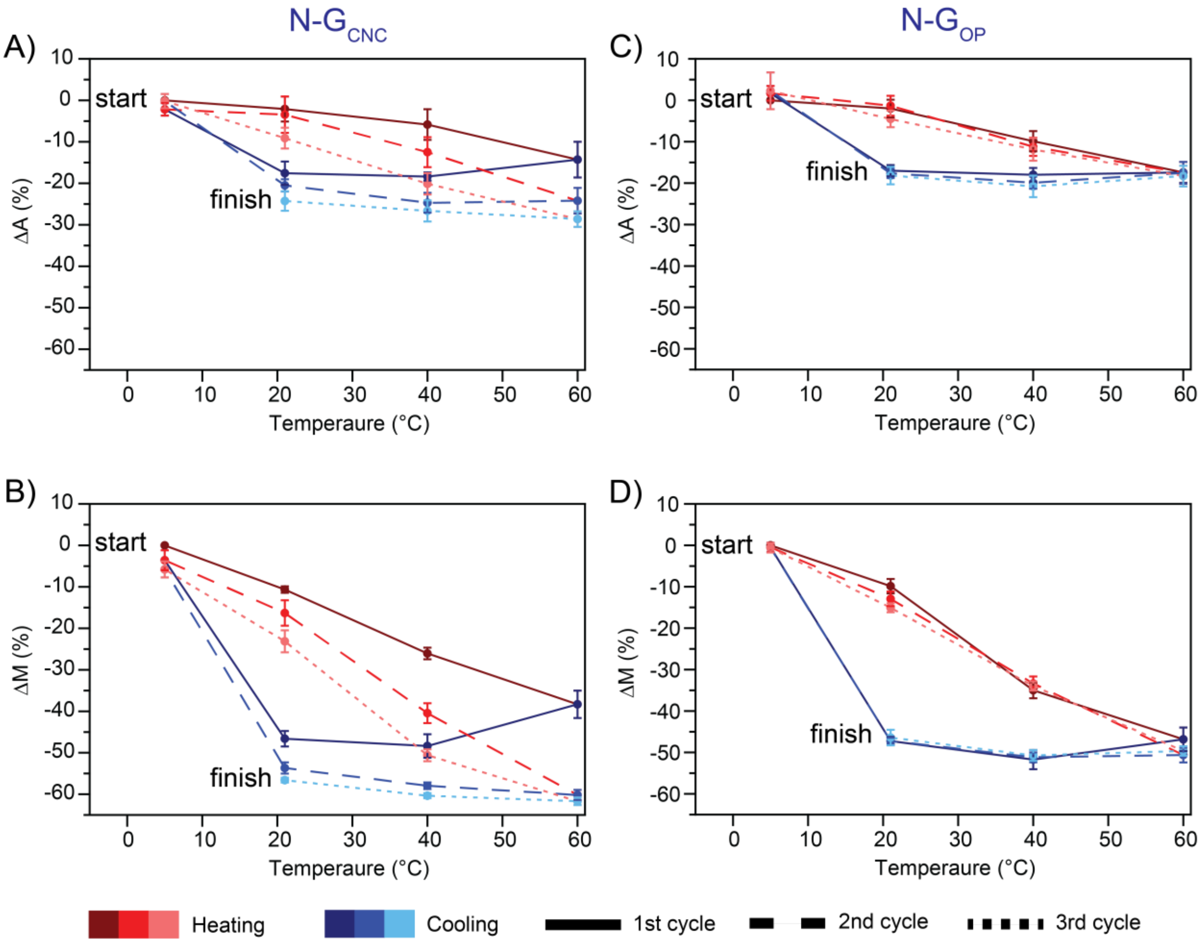

Figure S14. Temperature-dependent change in ellipsoidal area of solvent exchanged (A) **N-G$_{OP}$** and (B) **N-G$_{CNC}$**. Temperature-dependent change in mass for solvent exchanged (C) **N-G$_{OP}$** and (D) **N-G$_{CNC}$**.

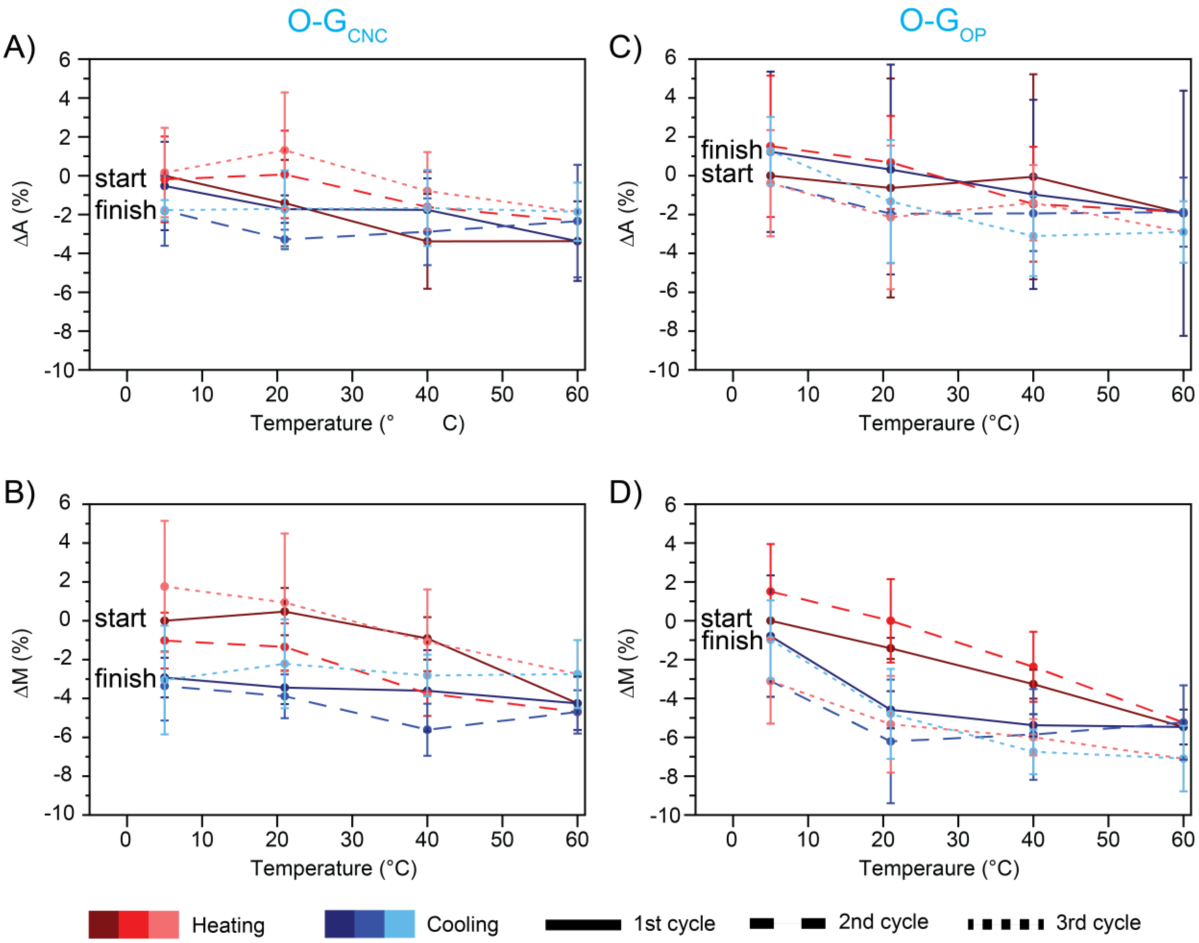

Figure S15. Temperature-dependent change in ellipsoidal area of solvent exchanged (A) **O-G_{CNC}** and (B) **O-G_{OP}**. Temperature-dependent change e in mass for solvent exchanged (C) **O-G_{CNC}** and (D) **O-G_{OP}**.

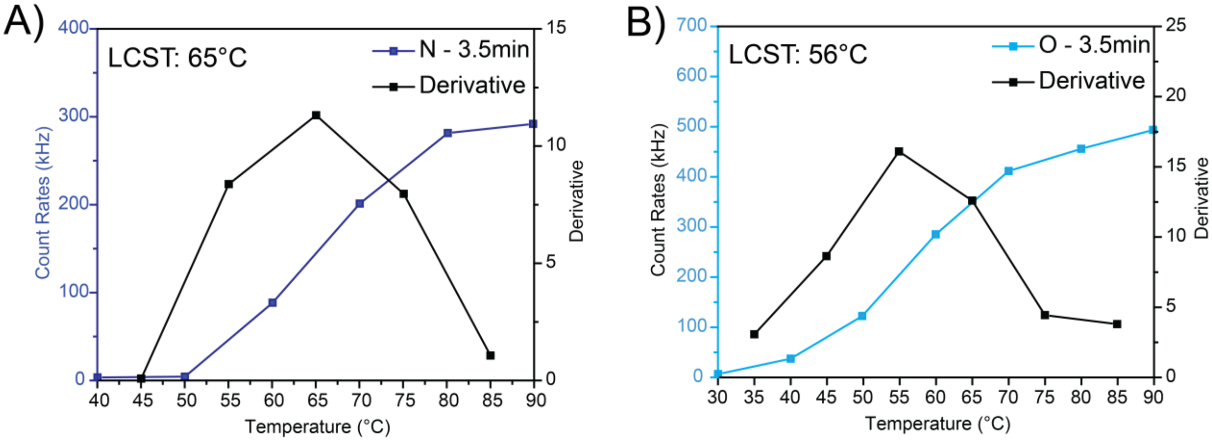

Figure S16. Diffusing-wave spectra of the free polymers **N** and **O** (i.e., no allyl groups) showing the scattering frequency versus temperature for 3 %w/w dispersions in MQ H_2O. (A) Spectrum of **N** overlayed with derivative. (B) Spectrum of **O** overlayed with derivative.

References

(S1) Majoinen, J.; Walther, A.; McKee, J. R.; Kontturi, E.; Aseyev, V.; Malho, J. M.; Ruokolainen, J.; Ikkala, O. Polyelectrolyte Brushes Grafted from Cellulose Nanocrystals Using Cu-Mediated Surface-Initiated Controlled Radical Polymerization. *Biomacromolecules* **2011**, *12* (8), 2997–3006. https://doi.org/10.1021/bm200613y.

(S2) Glasing, J.; Bouchard, J.; Jessop, P. G.; Champagne, P.; Cunningham, M. F. Grafting Well-Defined CO2-Responsive Polymers to Cellulose Nanocrystals via Nitroxide-Mediated Polymerisation: Effect of Graft Density and Molecular Weight on Dispersion Behaviour. *Polym. Chem.* **2017**, *8* (38), 6000–6012. https://doi.org/10.1039/c7py01258f.
